# NARO historical phenotype dataset from rice breeding

**DOI:** 10.1270/jsbbs.23040

**Published:** 2024-03-08

**Authors:** Kei Matsushita, Akio Onogi, Jun-ichi Yonemaru

**Affiliations:** 1 Research Center for Agricultural Information Technology (RCAIT), National Agriculture and Food Research Organization (NARO), 3-1-1 Kannondai, Tsukuba, Ibaraki 305-8517, Japan; 2 Institute of Crop Science (NICS), NARO, 2-1-2 Kannondai, Tsukuba, Ibaraki 305-8602, Japan; 3 Faculty of Agriculture, Ryukoku University, 1-5 Yokotani, Seta Oe-cho, Otsu, Shiga 520-2194, Japan

**Keywords:** rice, historical data, yield, release year, BLUP, PCA, NARO

## Abstract

Data from breeding, including phenotypic information, may improve the efficiency of breeding. Historical data from breeding trials accumulated over a long time are also useful. Here, by organizing data accumulated in the National Agriculture and Food Research Organization (NARO) rice breeding program, we developed a historical phenotype dataset, which includes 6052 records obtained for 667 varieties in yield trials in 1991–2018 at six NARO research stations. The best linear unbiased predictions (BLUPs) and principal component analysis (PCA) were used to determine the relationships with various factors, including the year of cultivar release, for 15 traits, including yield. Yield-related traits such as the number of grains per panicle, plant weight, grain yield, and thousand-grain weight increased significantly with time, whereas the number of panicles decreased significantly. Ripening time significantly increased, whereas the lodging degree and protein content of brown rice significantly decreased. These results suggest that panicle-weight-type high-yielding varieties with excellent lodging resistance have been selected. These trends differed slightly among breeding locations, indicating that the main breeding objectives may differ among them. PCA revealed a higher diversity of traits in newer varieties.

## Introduction

Rice is a staple food in Japan. Our ancestors tried to increase rice production by selecting plants that can achieve high yields and by improving cultivation methods while overcoming damage caused by pests, diseases, and abnormal weather conditions. As a result, many local cultivars have been established and used in each region in Japan. In the Meiji era, the modern breeding method using crosses and selection of progenies on the basis of genetics was introduced by the former National Agricultural Research Organization (NARO). ‘Rikuu 132’, which was released in 1921, was the first rice cultivar developed through modern breeding methods in Japan.

Many core cultivars of rice have been developed at the six regional NARO research centers located throughout Japan (ex. “Kinumusume” (Kaji *et al.* 2009), “Kinuhikari” (Koga *et al.* 1989)). From the 1960s onward, as the domestic demand for rice was satisfied, rice quality became more important than high yield. Therefore, the mainstream of rice breeding was changed to the development of cultivars with good eating quality that were derived from ‘Koshihikari’ (Kobae and The Miyazaki breeding group of rice 2005, Koga *et al.* 1989, Saito *et al.* 1989, Sasaki *et al.* 1990, 1993). Research on the relationship between eating quality and the physical and chemical properties of cooked rice has also progressed (Kurasawa *et al.* 1969, 1972), leading to the introduction of mutated genes that reduce amylose content with the aim of improving eating quality (Ando *et al.* 2007, Iida *et al.* 2011, Ise *et al.* 2001, Kunihiro *et al.* 1993, Sato *et al.* 2008). In recent years, the need to adapt to global warming has led to the breeding of cultivars with high ability to ripen under high-temperature conditions (Kaji *et al.* 2009, Nagaoka *et al.* 2020, Sakai *et al.* 2010, Sasahara *et al.* 2018a, Wakamatsu *et al.* 2016). The effect of high yield on the price of rice has also been recently reevaluated, and the breeding of cultivars with high yield potential has been resumed (Ando *et al.* 2011, Ohta *et al.* 2019, Sakai *et al.* 2014, Sasahara *et al.* 2018b, Sato *et al.* 2019).

The conversion of rice grown in surplus in paddy fields to forage was advocated (Iida and Takahashi 1976, Takahashi and Iida 1963, 1965), and the breeding of cultivars with high biomass for whole-crop silage has been underway (Kato 2008, Kato *et al.* 2010, Niwayama *et al.* 1989, Sakai *et al.* 1989, 2003, 2008). Short-panicle cultivars that are more suitable for roughage have become mainstream (Matsushita *et al.* 2011, 2014, Nakagomi *et al.* 2019), and cultivars with extremely high yields suitable for use as concentrate feed have also been bred (Goto *et al.* 2009, Hirabayashi *et al.* 2010, Miura *et al.* 2006, Nakagomi *et al.* 2006, 2017). During the breeding of high-yielding cultivars for food or feed, the high-yielding ability of Indica cultivars has been introduced.

In the breeding process, various breeding lines, such as local numbered lines that were not registered as cultivars, and various data have been accumulated through phenotypic evaluations, known as multi-environment trials (METs), in experimental fields. The main objective of METs is to assess the performance of breeding lines under different environmental conditions or in different locations, or both, and to check their ability to adapt to target environments. The analysis of historical data accumulated through METs provides information about the overall performance of each breeding line and its genotype by environment (G × E) interaction.

As mentioned above, NARO has successfully developed a diverse range of paddy rice cultivars, each exhibiting unique characteristics. The dataset derived from field performance tests conducted during the registration of these varieties includes valuable information, encompassing local numbered lines that have yet to be officially recognized as distinct varieties. This dataset holds immense potential as a valuable phenotypic resource for numerous genotypes, enabling a comprehensive exploration of trait changes throughout the breeding process and intricate relationships among traits.

Recently, with the development of high-throughput genotyping, there has been an increase in the use of historical data, sometimes obtained from many breeding lines, for genetic studies of potato (Lindqvist-Kreuze *et al.* 2014), barley (Gonzalez *et al.* 2018), durum wheat (Johnson *et al.* 2019), and maize (Dias *et al.* 2020). These historical data in combination with environmental information have been used to predict yield and other traits (Tack *et al.* 2017). Khanna *et al.* (2022), researchers of the International Rice Research Institute in the Philippines have estimated the genetic trends of grain yield under non-stress and drought conditions, and also by using combined data from more than one environmental condition. In Japan, historical data on soybean breeding over the past 50 years have been collected, organized, and used to clarify the relationship between the environment and agricultural traits (Onogi *et al.* 2021).

In this study, we produced a NARO rice historical phenotype dataset that includes 6052 records for 667 varieties obtained in yield trials from 1991 to 2018 at six NARO research stations. To clarify the changes in 15 traits, including yield, according to the cultivar release year of breeding, we used the best linear unbiased predictions (BLUPs) (Piepho *et al.* 2008) and principal component analysis (PCA). The obtained results were utilized to explore the alterations in traits within domestic rice breeding, aiming for genetic enhancements such as increased yield and quality. Additionally, the study delved into various plant types and the potential changes that could be inferred from multiple traits.

## Materials and Methods

### Construction of NARO rice historical phenotype dataset

We assembled documents written about bred lines in 1991–2018 at six NARO research stations (see the abbreviation for each station in Table 1). The dataset was developed by using summarized information on yield trials from these documents. Most of the documents are stored as electronic files; printed records were digitized by using optical character recognition. In all yield trials, several check varieties were used together with the elite lines. Most of the check varieties were registered or common cultivars, including a few other genetic resources. The developed historical phenotype dataset contained 6052 records for 667 varieties (Table 1). No variety was examined at all six stations; 507 varieties were each examined at only one research station; 119 at two, 25 at three, 14 at four, and 2 varieties at five research stations. Most varieties were examined over many years under different conditions. The mean of the number of records per variety was 9.073 and the mode was 3 (Supplemental Fig. 1).

Items in each record were classified into cultivation methods and trait descriptions. Cultivation methods consisted of four types of categorical data (variety name, experimental field, cropping season [1: early, 2: mid-early, 3: mid-late, 4: late], and planting method) and seven types of numerical data (year, seeding date, transplanting date, planting density, amount of nitrogen in basal dressing, frequency of topdressing, and amount of nitrogen in topdressing) (Supplemental Tables 1–4). Fifteen agronomic traits are listed in Table 2. Data sufficiency of all traits except the amylose content of milled rice and the protein content of brown rice ranged from 78% to 97% at all stations. At HARC, at least 50% of all records included the amylose content of milled rice and protein content of brown rice; in contrast, ≤24% of records at the other stations included these data. Units of numeric traits were adjusted among data and clearly clerical errors were deleted. The planting density in direct seeding, expressed as seed number per unit area, was recalculated as weight per unit area assuming that the weight of a seed was 25 mg. Days to heading and days to maturity were calculated by subtracting the seeding date from the heading and maturity dates, respectively. The number of grains per panicle was calculated by dividing grain yield by the number of panicles and grain weight. Harvest index was calculated by dividing grain yield by whole plant weight. Lodging degree was scored from 0 (erect plants) to 5 (plants with lodging). Grain quality was classified by appearance into nine grades, from 1 (excellent) to 9 (very bad). This dataset has the potential to be shared with researchers from external institutions, adhering to NARO’s intellectual property policy.

### Statistical analysis

To estimate BLUPs for each trait, the dataset was arranged to simplify the mixed model. We used only those data from early transplanting at NICS but the data from transplanting at the standard time at the other five sites (Supplemental Table 1). As a result of further processing of missing values to other parameters, out of 6052 records for 667 varieties, 4555 records for 626 varieties were used (Supplemental Table 5). The general four-way mixed model was adopted to estimate the random effects of genotypes on 15 agronomic traits (Table 2). To select the appropriate model for 13 agronomic traits (with amylose content of milled rice and protein content of brown rice excluded), we created a model incorporating two fixed effects (manure levels, expressed as the total amount of nitrogen in basal and topdressings, and location), two random effects (genotypes and years), and all their interactions, and we selected the best model for each of the 13 traits by using the step function of the R-package lmerTest (Kuznetsova *et al.* 2017). The common model (1) was constructed with the effects and interaction terms selected for at least 10 of the 13 traits (Supplemental Table 6).

$$\begin{array}{ll} y_{\text{ijkm}}= & \mu +G_{i}+L_{j}+Y_{k}+F_{m}+{\text{GL}}_{\text{ij}}+{\text{LY}}_{\text{jk}}\\ & +{\text{LM}}_{\text{jm}}+{\text{GLY}}_{\text{ijk}}+{\text{LYF}}_{\text{jkm}}+\varepsilon_{\text{ijkm}} \end{array}$$ (1)

where yield *y_ijkm_* was determined for *i* genotypes at *j* locations over *k* years with *m* manure levels. The effects of manure level (*F_m_*) and location (*L_j_*) were treated as fixed, whereas the effects of genotype (*G_i_*), year (*Y_k_*), and the interaction terms of genotype-by-location (*GL_ij_*), location-by-year (*LY_jk_*), location-by-manure level (*LF_jm_*), genotype-by-location-by-year (*GLY_ijk_*), location-by-year-by-manure level (*LYF_jkm_*), and the error *ε_ijkm_* were assumed to be independent and to have constant variances over the levels of effects.

For the amylose content of milled rice and the protein content of brown rice, we used model (2) because the number of data was unbalanced by location:

$$y_{\text{ijk}}=\mu +G_{i}+Y_{j}+F_{k}+{\text{GY}}_{\text{ij}}+\varepsilon_{\text{ijk}}$$ (2)

The BLUPs of random effects were extracted by using the ranef function in the R library lmer (Bates *et al.* 2015). Changes in 11 agronomic traits (days to maturity, harvest index, amylose content of milled rice and protein content of brown rice were excluded due to high collinearity with other traits or unbalanced data) during the breeding periods for all varieties were analyzed by PCA of BLUPs for genotype effects using the princomp function in R software. The values of principal components and their loading were used to visualize trait changes of varieties with different release years.

To consider the possible bias effects of uniformly considering different diverse populations, we also created the “NARO-variety-dataset” which contains data for 416 cultivars and varieties bred at only NARO from 1991 to 2018 and performed the same analysis.

## Results

### Cultivation methods in the NARO rice historical phenotype dataset

Transplanting was the major planting method in the dataset (Supplemental Table 1). The proportion of direct seeding in all trials was 13% in flooded paddy fields and 3% in well-drained paddy fields. At the three northern stations—HARC, TARC, and CARC—transplanting was performed in mid- or late May and was classified as standard transplanting (Supplemental Table 1). Similar transplanting dates were classified as early at NICS, WARC, and KARC. The main transplanting dates at WARC were in early or mid-June and were classified as standard. Transplanting in mid- or late June was classified as late at TARC, CARC, and NICS, and as standard at KARC. Late transplanting at TARC was used very rarely.

In trials that used transplanting, the mode of planting density (hills/m^2^) was 24.0 at HARC, 22.2 at TARC and NICS, 20.8 at WARC and KARC, and 18.5 at CARC (Supplemental Table 2). Trials with the lowest minimum planting density were found at the three northern stations, HARC (13.3), TARC (11.1), and CARC (11.1). The mode of planting density in trials that used direct seeding was 10 g/m^2^ at HARC—much higher than at the other stations (3–5 g/m^2^).

The lowest minimum amount of total nitrogen applied was 4 g/m^2^, and the highest maximum amount was 29 g/m^2^ (Supplemental Table 3). The mode of total nitrogen applied (g/m^2^) was 10 at HARC, 8 at NICS and KARC, 7 at TARC and WARC, and 6 at CARC. Nitrogen application at basal dressing ranged from 3 to 24 g/m^2^ and the mode ranged from 4 to 10 g/m^2^.

### Nine traits in the dataset

**Fig. 1.**
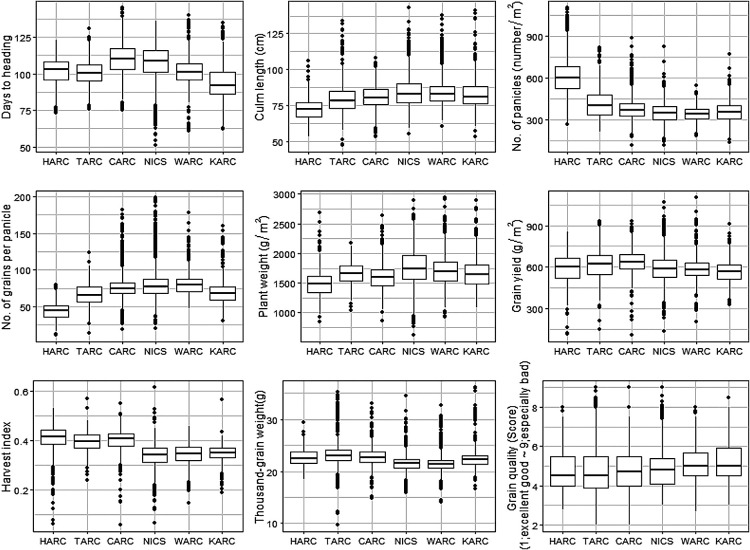
Box plots of nine agronomic traits at six research stations

We plotted phenotypes of nine traits, namely days to heading, culm length, number of panicles, number of grains per panicle, plant weight, grain yield, harvest index, thousand-grain weight, and grain quality (Fig. 1). The overall mean of days to heading was 103, and the means were 109 at CARC, 107 at NICS, 102 at WARC, 101 at HARC, 100 at TARC, and 93 at KARC. Days to heading was greater at CARC and NICS than at HARC and TARC, which are located in cooler environments. The mean culm length at HARC was 72 cm—shorter than at the other stations (80–84 cm). Mean number of panicles was greater at HARC than at the other stations, whereas the mean number of grains per panicle and plant weight were lower. Mean grain yield (g/m^2^) was 638 at CARC, 623 at TARC, 590 at HARC, 587 at NICS, 586 at WARC, and 566 at KARC. Mean harvest index was 0.39–0.40 at the three northern stations and 0.34–0.35 at the three southern stations. Mean thousand-grain weight was 22.7 at HARC, 23.6 at TARC, 22.9 at CARC, 21.6 at NICS, 21.6 at WARC, and 22.6 at KARC. Mean grain quality score ranged from 4.8 to 5.2. The data for other traits are shown in Supplemental Fig. 2.

### Trait changes during the breeding period

**Fig. 2.**
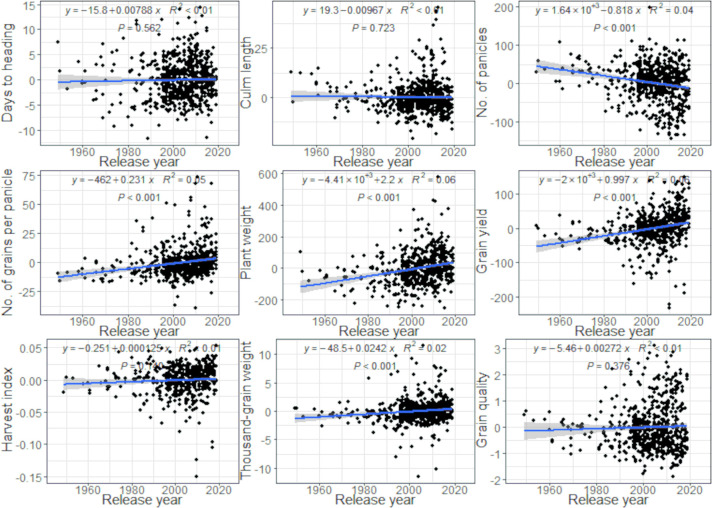
Changes in BLUPs for genotype effects on nine agronomic traits (all dataset). Each black symbol corresponds to a variety.

We plotted the changes in BLUPs for genotype effects on nine traits (days to heading, culm length, number of panicles, number of grains per panicle, plant weight, grain yield, harvest index, thousand-grain weight, and grain quality) during the past seven decades (Fig. 2). Significant increases were observed in the number of grains per panicle (*R*^2^ = 0.05, *P* < 0.001), plant weight (*R*^2^ = 0.06, *P* < 0.001), grain yield (*R*^2^ = 0.06, *P* < 0.001), and thousand-grain weight (*R*^2^ = 0.02, *P* < 0.001). The data for other traits are shown in Supplemental Fig. 3. Number of panicles was the only parameter that decreased significantly (*R*^2^ = 0.04, *P* < 0.001).

**Fig. 3.**
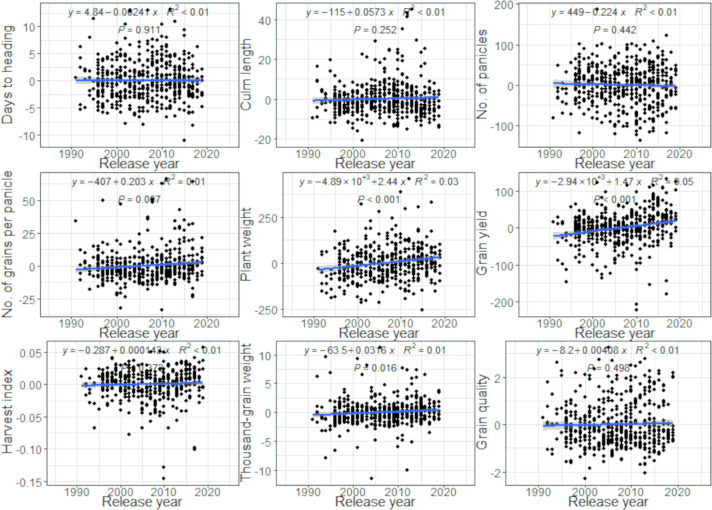
Changes in BLUPs for genotype effects on nine agronomic traits (NARO-variety-dataset). Each black symbol corresponds to a variety.

The results of the analysis using the “NARO-variety-dataset” are also shown in Fig. 3 and Supplemental Fig. 4. Although the short period and the small number of varieties, significant increases were observed in the number of grains per panicle (*R*^2^ = 0.01, *P* < 0.007), plant weight (*R*^2^ = 0.03, *P* < 0.001), grain yield (*R*^2^ = 0.05, *P* < 0.001), and thousand-grain weight (*R*^2^ = 0.01, *P* < 0.016) as in the results using all data (Fig. 2).

To identify changes in BLUPs for genotype at each location, we examined the associations between the BLUPs for 13 traits of those varieties bred at each research station of NARO using the “NARO-variety-dataset” and the release year as in Table 3. The ripening period was significantly lengthened at TARC (*R*^2^ = 0.08, *P* < 0.05). At KARC, the number of grains per panicle (*R*^2^ = 0.11, *P* < 0.01) increased, but the number of panicles (*R*^2^ = 0.09, *P* < 0.01) decreased. This trend was similar to the decrease in the number of panicles and increase in the number of grains per panicle in the overall data mentioned above (Fig. 2). Significant increase in grain yield was observed in four stations (HARC, TARC, CARC, and NICS). Thousand-grain weight significantly increased at both HARC (*R*^2^ = 0.14, *P* < 0.001) and WARC (*R*^2^ = 0.06, *P* < 0.05). Lodging degree decreased at WARC (*R*^2^ = 0.20, *P* < 0.001).

**Fig. 4.**
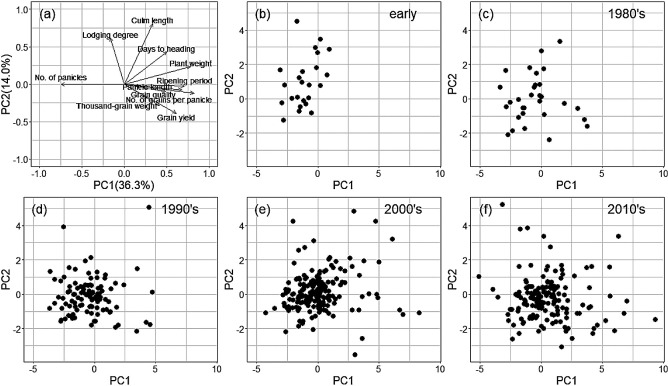
PCA plots for the first two principal components obtained from BLUPs for genotype effects on 11 agronomic traits (all data). (a) Loading plot shows how strongly each characteristic influenced a principal component. (b) - (e) Biplot shows the variability of the first two principal components among varieties bred in early, 1980’s, 1990’s, 2000’s, and 2010’s year, respectively.

The PCA and the principal components with dimensional reduction of trait information allowed us to visualize the relationships among multiple traits (Fig. 4). We considered PC1, which explained by 36.3% of all the traits, to be related to crop biomass because of a large difference in plant weight and number of panicles among the 11 traits used (Fig. 4a). PC2 (14.0% of the total) may be related to grain yield, as it varied between grain yield and culm length. The following traits were closely related to each other: grain yield and thousand-grain weight; lodging degree and culm length; grain quality, panicle length, ripening period, and number of grains per panicle. The results of PC1 and PC2 for all varieties separated by breeding periods showed that many of the varieties had similar distributions (Fig. 4b–4f). Varieties bred before 1980 had long culms and severe lodging (Fig. 4b), but those bred after 2000 had a variety of different characteristics, including high grain yield (Fig. 4e, 4f).

To characterize varieties bred at each location, we also examined PCA analysis of varieties bred at each research station of NARO using the “NARO-variety-dataset” (Supplemental Fig. 5). The directions of factor loadings showed patterns similar to that in Fig. 4a. That is, number of panicles showed opposite or nearly orthogonal directions to the other traits.

## Discussion

Considering the only research stations that showed regression lines with a significant slope in BLUPs for 13 agronomic traits during the release years of varieties, the positive slope of grain yield at different locations can be attributed to the breeding and selection process, which optimized the balance of yield-related traits to adapt the growth environment (Table 3). Varieties bred at all locations tended to have fewer panicles and more grains per panicle, suggesting that panicle-weight-type varieties were bred more frequently during rice breeding (Fig. 2). The number of panicles tended to decrease as grain yield increased, whereas the number of grains per panicle and thousand-grain weight tended to increase in Japan (Fig. 2).

The origin of the high-yielding ability can be understood by analyzing genealogical information using the database published by NARO (Kajiya-Kanegae *et al.* 2022). In the 1980s, ‘Akenohoshi’ (Shinoda *et al.* 1989) and ‘Habataki’ (Miura *et al.* 1991) were bred as descendants of the sibling lines derived from an Indica/Japonica hybrid crossing. Following this, a multitude of high-yielding rice cultivars, essential for staple food and forage purposes, emerged from the Korean hybrid group known as the Tongil type (Chung and Heu 1991). The Tongil type is distinguished by its high number of grains per panicle, substantial sink capacity, semi-dwarf stature, and notable resistance to lodging. However, this group of cultivars has a poor low-temperature tolerance, as shown at cold locations such as TARC, and similar high-yield characteristics are often introduced from ‘Fukuhibiki’ (Higashi *et al.* 1994). This cultivar and its descendants have also been used to breed cultivars for whole-crop silage due to their superior biomass production (Kato 2008). In addition, the US cultivar ‘Lemont’ has been used to breed the large-biomass–producing forage cultivar ‘Tachiaoba’ (Sakai *et al.* 2008) and the good-tasting, high-yielding cultivar ‘Tachiharuka’ (Sakai *et al.* 2014).

At HARC, the observed characteristics included shorter culms, higher panicle numbers, shorter panicle lengths, and a lower number of grains per panicle. Similar trends were also observed at TARC (Fig. 1, Supplemental Fig. 2). In the analysis of the F_2_ population derived from a cross between “Kitaibuki” and “Akage”, it was noted that individuals with later days to heading exhibited a decrease in the number of panicles. Additionally, these individuals had longer panicle lengths, culm lengths, and a larger total number of grains (Fujino 2020). This trend aligns with general patterns observed in cold climates characterized by short growing seasons. The relatively small variability of yield components such as the number of grains per panicle and thousand-grain weight at HARC may be related to the lack of breeding of whole-crop silage cultivars (Fig. 1).

In both the Kanto region (NICS) and western Japan (WARC and KARC), a significant increase in plant weight and deterioration of grain quality were observed, whereas in northern Japan (HARC and TARC) and the Hokuriku region (CARC), a tendency toward higher grain yield was noted (Fig. 1). These observations suggest a correlation between these three traits and the temperature during the growth period. Previous studies have indicated that high temperatures during the ripening period can impact the apparent quality of brown rice (Morita *et al.* 2002). Therefore, it is likely that the observed correlation with locations is influenced by temperature variations.

The relationships among the traits varied across the different locations (Supplemental Fig. 5). In Hokkaido and Tohoku, plant weight exhibited a similar pattern to the ripening period, whereas in locations southwest of CARC, plant weight showed a similar trend to the days to heading. Plant weight could be influenced by the duration of the growing season, and in warmer regions, it could be influenced by the days to heading. Utilizing plant weight metrics throughout the growth process may provide additional insights into the intricate relationship between genetics and the environment in plant growth. At CARC, grain yield and plant weight increased with time (Table 3); this may have increased both sink and source capacity, thus increasing grain yield.

The first step of high yields in modern rice breeding began with the use of a semi-dwarf gene to suppress the lodging associated with high nitrogen application. A high-yielding semi-dwarf variety “IR8” derived from the “Dee-Geo-Woo-Gen”, which possesses a nonfunctional *sd1* gene (Sasaki *et al.* 2002) was bred by the International Rice Research Institute (IRRI) in the early 1960s (Khush 1999). In Japan, “Reimei” (Futsuhara *et al.* 1967), which has a nonfunctional *sd1* gene induced by radiation mutation, and descendent varieties derived from it are also used. The New-Plant-Type (NPT) varieties started to be bred at IRRI and in China in the early 1990s (Peng *et al.* 2008). The breeding of NPT varieties, which are characterized by few tillers and a heavy panicle weight, is one of the ideal plant types for high-yielding rice and affected the selection of panicle-weighted type varieties in Japan.

Nitrogen fertilization to increase plant weight increases the protein content in brown rice, leading to a decrease in eating quality (Balindong *et al.* 2018), and therefore, selection to reduce the protein content in brown rice has been conducted in Japan. High-yield breeding using hybrid rice varieties (Bai *et al.* 2018), which has been done in China, has not been done much domestically due to problems with hybrid seed production. In recent years, molecular breeding, focusing on a pyramiding of useful genes using genomic information related to yield, has been progressing both domestically and internationally. In the future, we will use genomic information to study genetic and phenotypic changes during rice breeding in Japan.

## Author Contribution Statement

KM and JY designed the experiments. AO and JY analyzed the data and interpreted the results. KM and JY wrote the paper. All authors read and revised the paper.

## Supplementary Material

Supplemental Figures

Supplemental Tables

## Figures and Tables

**Table 1.** Characteristics of the historical rice phenotype dataset of NARO

Research station (location of experimental field)	Latitude*^a^*	Longitude*^a^*	Years	No. of verieties	No. of records
Hokkaido Agricultural Research Center (Sapporo, Hokkaido), HARC	43.0085	141.41144	1995–2018	77	659
Tohoku Agricultural Research Center (Daisen, Akita), TARC	39.4913	140.49638	1992–2018	144	813
Central Region Agricultural Research Center (Joetsu, Niigata), CARC	37.1164	138.27047	1991–2018	185	1216
Institute of Crop Science (Tsukubamirai, Ibaraki), NICS	36.0090	140.02201	1994–2018	181	1639
Western Region Agricultural Research Center (Fukuyama, Hiroshima), WARC	34.5017	133.38432	1992–2018	155	867
Kyushu Okinawa Agricultural Research Center (Chikugo, Fukuoka), KARC	33.2079	130.49108	1994–2018	144	858
				886 (667)*^b^*	6052

*^a^* Position of Agro-Meteorological Grid Square *^b^* The number in parentheses is the total number of non-redundant varieties among the research stations.

**Table 2.** Numbers of records for 15 agronomic traits at the six research stations

Trait	Unit	HARC	TARC	CARC	NICS	WARC	KARC	Total	Data sufficiency*^a^*
Days to heading*^b^*	days	619 (40)	790 (23)	1186 (30)	1614 (25)	847 (20)	802 (56)	5858 (194)	97%
Days to maturity*^c^*	days	590 (69)	768 (45)	1115 (101)	1592 (47)	743 (124)	713 (145)	5521 (531)	91%
Ripening period*^d^*	days	602 (57)	765 (48)	1124 (92)	1597 (42)	750 (117)	716 (142)	5554 (498)	92%
Culm length	cm	629 (30)	810 (3)	1195 (21)	1624 (15)	855 (12)	829 (29)	5942 (110)	98%
Panicle length	cm	629 (30)	790 (23)	1195 (21)	1624 (15)	844 (23)	829 (29)	5911 (141)	98%
Number of panicles	Number/m^2^	629 (30)	800 (13)	1195 (21)	1619 (20)	851 (16)	832 (26)	5926 (126)	98%
Number of grains per panicle*^e^*	—	468 (191)	668 (145)	1113 (103)	1580 (59)	721 (146)	694 (164)	5244 (808)	87%
Plant weight	g/m^2^	609 (50)	204 (609)	1112 (104)	1530 (109)	796 (71)	795 (63)	5046 (1006)	83%
Grain yield	g/m^2^	471 (188)	685 (128)	1117 (99)	1597 (42)	793 (74)	700 (158)	5363 (689)	89%
Harvest index*^f^*	—	468 (191)	198 (615)	1083 (133)	1502 (137)	778 (89)	680 (178)	4709 (1343)	78%
Thousand-grain weight	g	589 (70)	754 (59)	1127 (89)	1598 (41)	748 (119)	721 (137)	5537 (515)	91%
Grain quality	Score 1 (good) to 9 (bad)	565 (94)	736 (77)	1121 (95)	1590 (49)	785 (82)	695 (163)	5492 (560)	91%
Lodging degree	Score 0 (erect) to 5 (lodging)	626 (33)	800 (13)	1193 (23)	1615 (24)	836 (31)	823 (35)	5893 (159)	97%
Amylose content of milled rice	%	348 (311)	58 (755)	277 (939)	72 (1567)	41 (826)	186 (672)	982 (5070)	16%
Protein content of brown rice	%	411 (248)	83 (730)	286 (930)	43 (1596)	26 (841)	173 (685)	1022 (5030)	17%

Value in parenthesis shows the number of missing data. *^a^* (Total number of data – number of missing data)/total number of data *^b^* Period from seeding to heading *^c^* Period from seeding to maturity *^d^* Period from heading to maturity *^e^* Number of grains per panicle = grain yield × 1000/(panicle number × thousand-grain weight) *^f^* Harvest index = grain yield/plant weight

**Table 3.** Significant changes in BLUPs for genotypes during the release years of varieties at each of NARO research stations

Trait	Research station*^a^*	*b^b^*	Std. error of *b*	*a^c^*	Std. error of *a*	t-value	*P*	*R* ^2^
Ripening period	**TARC**	**–83.78**	**37.86**	**4.17E–02**	**1.89E–02**	**2.21**	**2.99E–02**	**0.05**
Number of panicles	*KARC*	*3002.85*	*1133.65*	*–1.50E+00*	*5.65E–01*	*–2.65*	*9.78E–03*	*0.09*
Number of grains per panicle	**KARC**	**–861.30**	**299.47**	**4.29E–01**	**1.49E–01**	**2.87**	**5.36E–03**	**0.11**
Plant weight	**CARC**	**–7147.93**	**2497.60**	**3.56E+00**	**1.24E+00**	**2.86**	**5.07E–03**	**0.07**
Grain yield	**HARC**	**–3096.03**	**1440.63**	**1.54E+00**	**7.18E–01**	**2.15**	**3.73E–02**	**0.10**
	**TARC**	**–4809.76**	**1534.14**	**2.40E+00**	**7.65E–01**	**3.14**	**2.43E–03**	**0.11**
	**CARC**	**–3387.97**	**1316.09**	**1.69E+00**	**6.56E–01**	**2.58**	**1.13E–02**	**0.06**
	**NICS**	**–3134.19**	**1317.08**	**1.56E+00**	**6.56E–01**	**2.38**	**1.92E–02**	**0.05**
Thousand-grain weight	**HARC**	**–137.73**	**48.44**	**6.86E–02**	**2.41E–02**	**2.84**	**6.41E–03**	**0.14**
	**WARC**	**–125.03**	**55.49**	**6.23E–02**	**2.77E–02**	**2.25**	**2.75E–02**	**0.06**
Lodging degree	*WARC*	*42.91*	*10.01*	*–2.14E–02*	*4.99E–03*	*–4.28*	*5.38E–05*	*0.20*

*^a^* Boldface and Italic show a significant positive and negative slope (*P* < 0.05) of the regression line (y = a*x* + b), respectively. *^b^* Intercept of the regression line (y = a*x* + b) *^c^* Slope of the regression line (y = a*x* + b)
