# Supplementary material for: NARO historical phenotype dataset from rice breeding
Source: Breed Sci. 2024 Mar 8;74(2):114–23. doi: 10.1270/jsbbs.23040 (PMC11442108; doi:10.1270/jsbbs.23040)
Supplement: Supplementary file 1 — Supplemental Figures [file 74_114_s1.pdf]

doi: 10.1270/jsbbs.23040

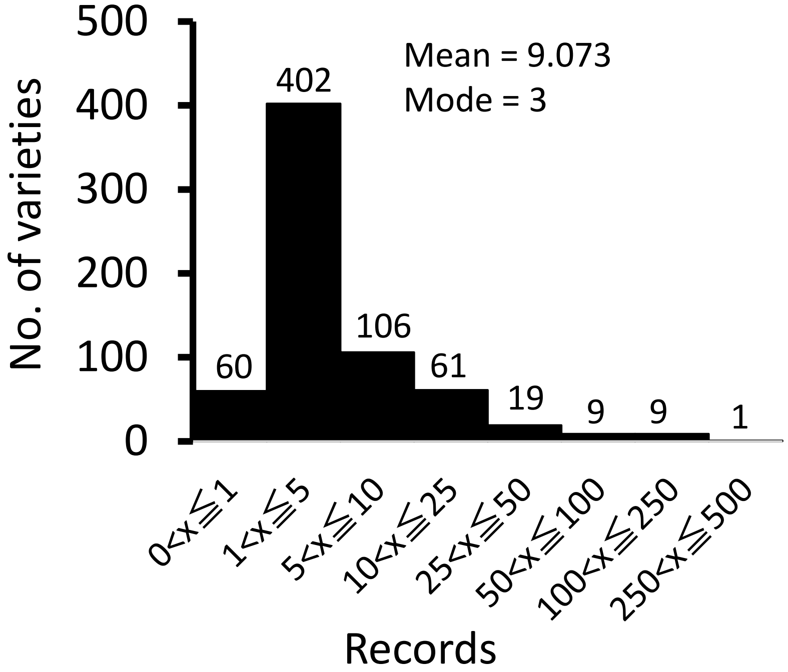


**Supplemental Fig. 1.** Number of records per variety.

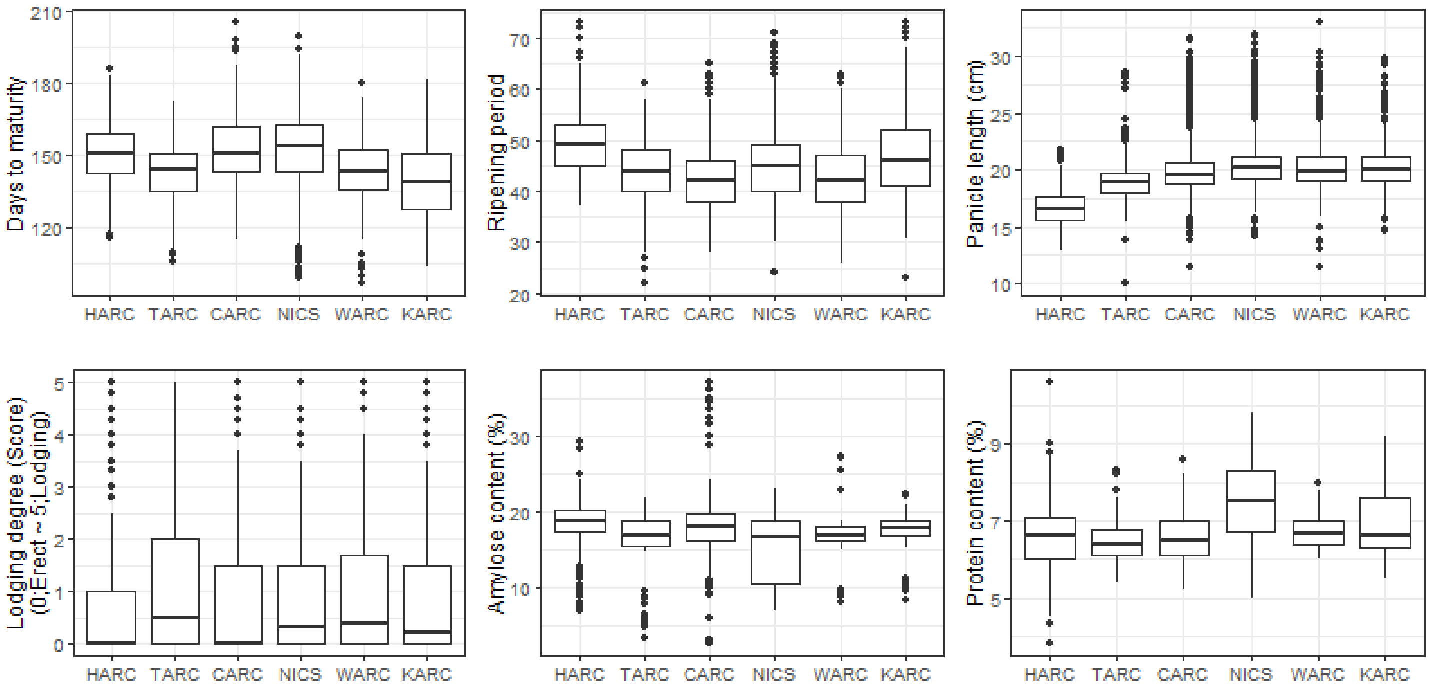


**Supplemental Fig. 2.** Box plots of six agronomic traits at the six research stations.

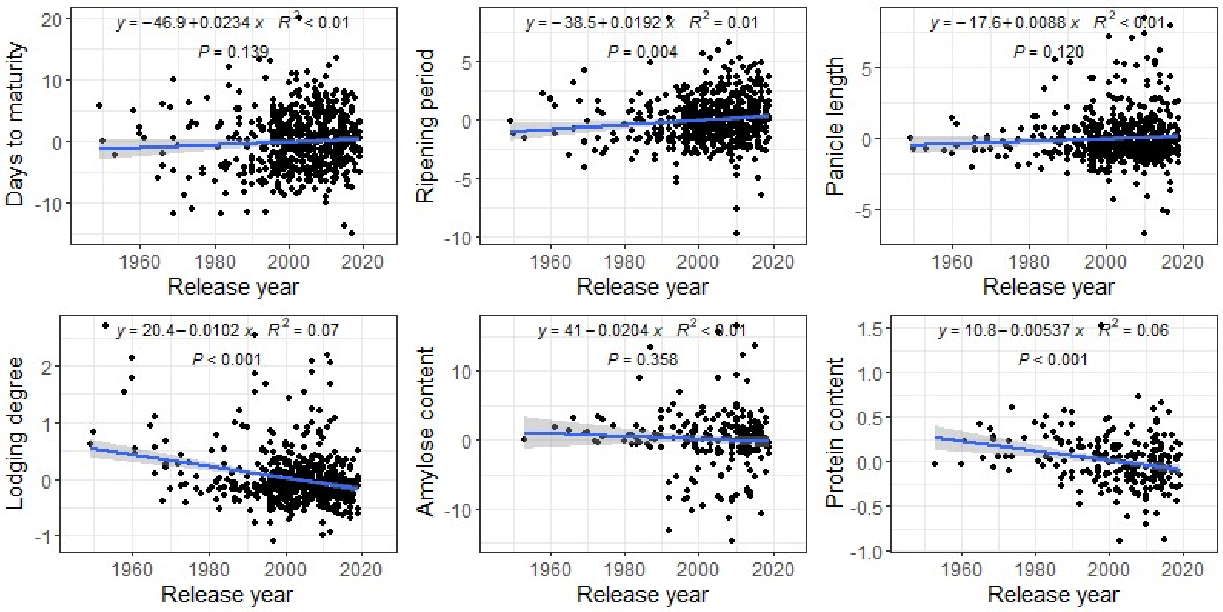


**Supplemental Fig. 3.** Changes in BLUPs for genotype effects on six agronomic traits (all data). Each black symbol corresponds to a variety.

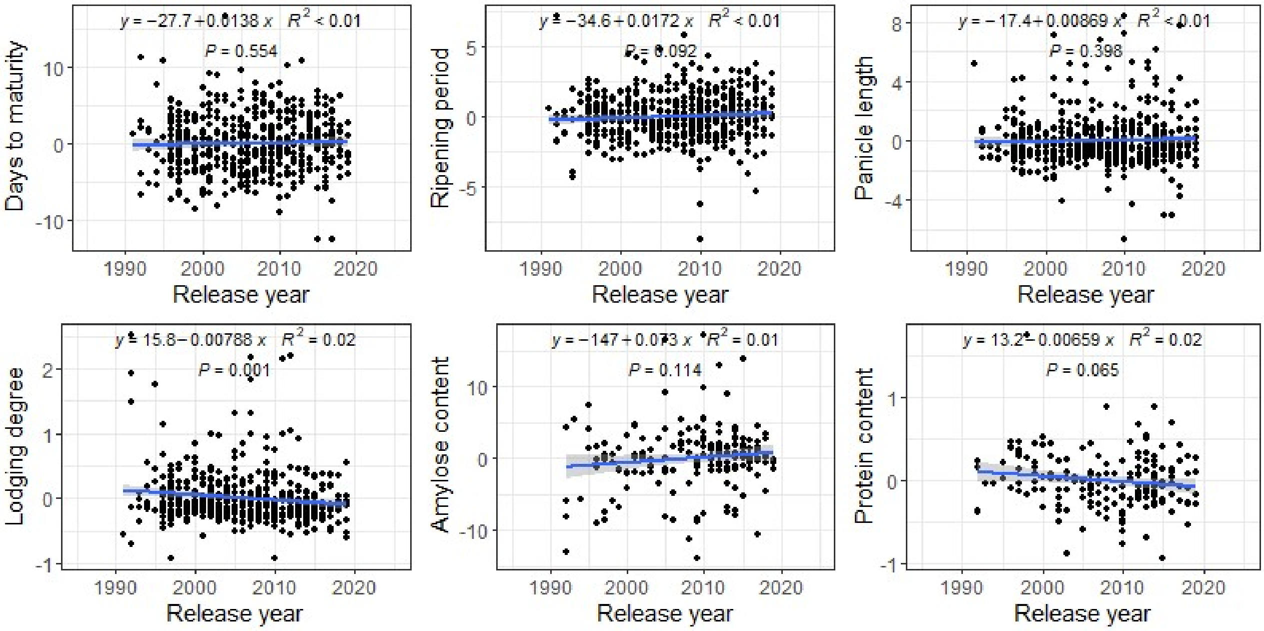


**Supplemental Fig. 4.** Changes in BLUPs for genotype effects on six agronomic traits (NARO-variety-dataset). Each black symbol corresponds to a variety.

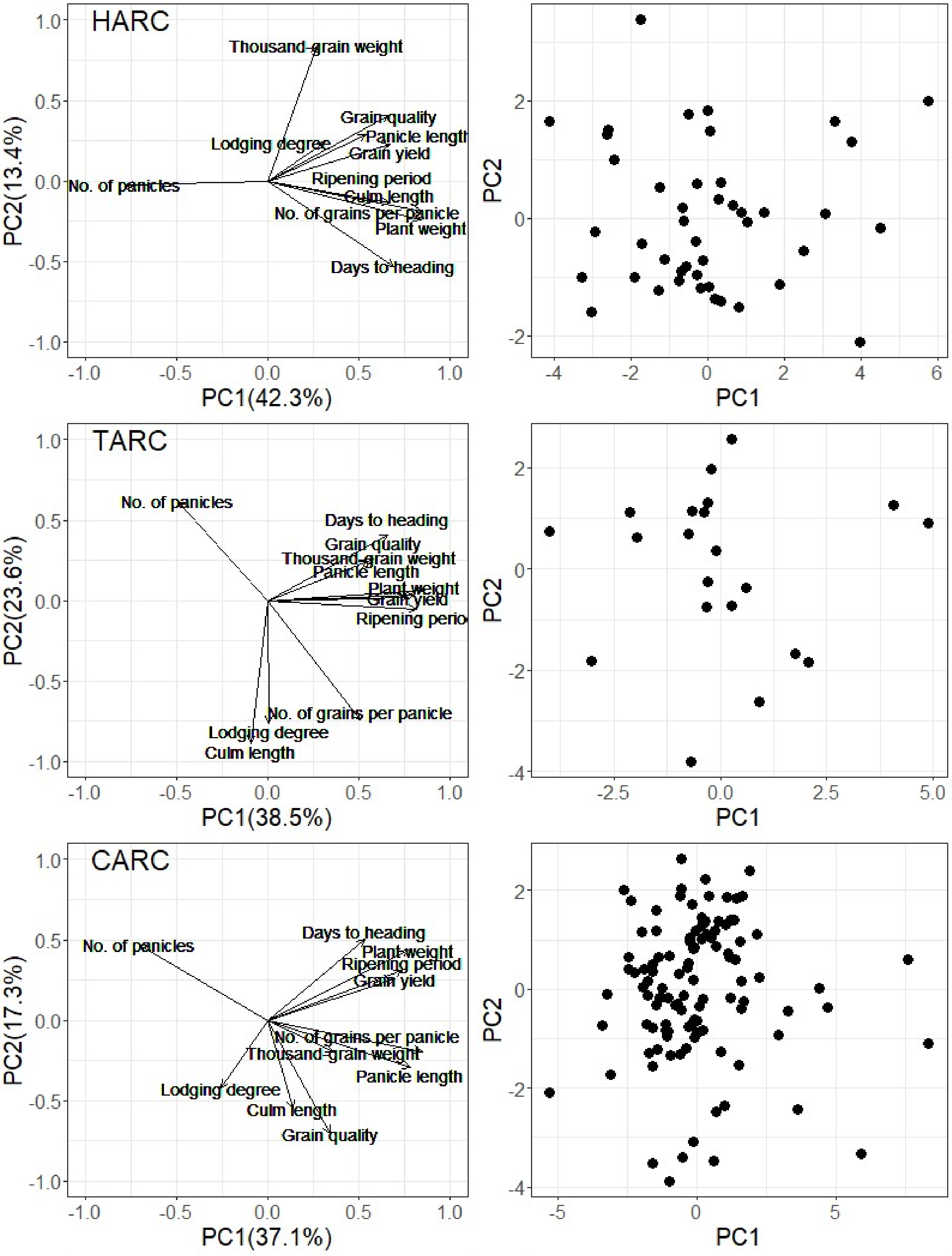
HARC
Thousand-grain weight
Grain quality
Panicle lengt
Lodging degree
Grain yield
No. of panicles
Ripening period
Culm length
No. of grains per panicle
Plant weight
Days to heading
PC2(13.4%)
PC1(42.3%)
TARC
No. of panicles
Days to heading
Grain quality
Thousand-grain weight
Panicle length
Plant weight
Grain yield
Ripening perio
No. of grains per panicle
Lodging degree
Culm length
PC2(23.6%)
PC1(38.5%)
CARC
No. of panicles
Days to heading
Plant weight
Ripening period
Grain yield
No. of grains per panicle
Thousand-grain weight
Panicle length
Lodging degree
Culm length
Grain quality
PC2(17.3%)
PC1(37.1%)
PC2
PC1

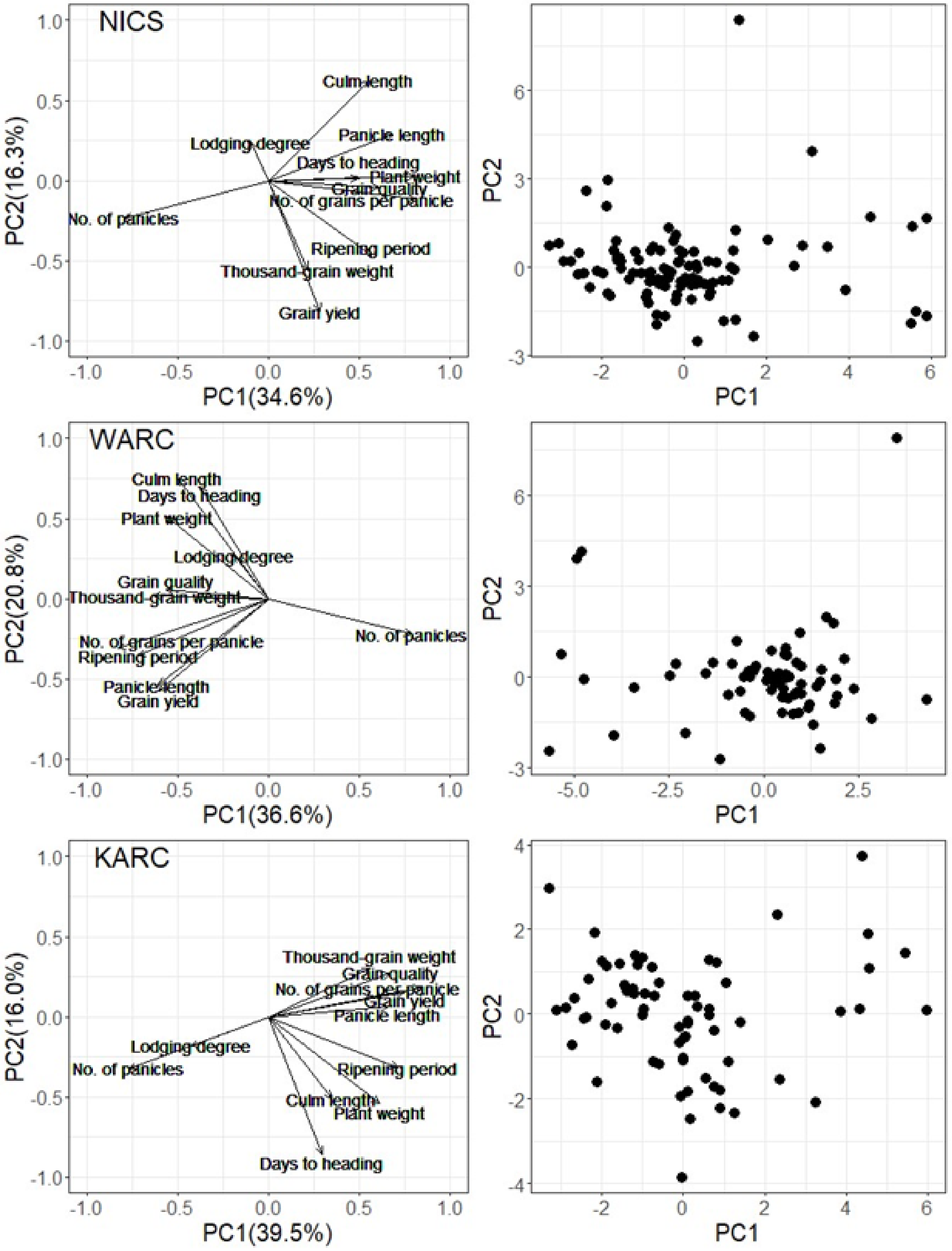
NICS
Culm length
Panicle length
Lodging degree
Days to heading
Plant weight
Grain quality
No. of grains per panicle
No. of panicles
Ripening period
Thousand-grain weight
Grain yield
PC2(16.3%)
PC1(34.6%)
PC2
PC1
WARC
Culm length
Days to heading
Plant weight
Lodging degree
Grain quality
Thousand-grain weight
No. of grains per panicle
Ripening period
No. of panicles
Panicle length
Grain yield
PC2(20.8%)
PC1(36.6%)
PC2
PC1
KARC
Thousand-grain weight
Grain quality
No. of grains per panicle
Grain yield
Panicle length
Lodging degree
No. of panicles
Ripening period
Culm length
Plant weight
Days to heading
PC2(16.0%)
PC1(39.5%)
PC2
PC1

**Supplemental Fig. 5**. PCA plots for the first two principal components obtained from BLUPs for genotype effects in varieties bred at each NARO research station on 11 agronomic traits (NARO-variety-dataset). Right: Loading plot shows how strongly each characteristic influenced a principal component. Left: Biplot shows the variability of the first two principal components.
