# Supplementary material for: NARO historical phenotype dataset from rice breeding
Source: Breed Sci. 2024 Mar 8;74(2):114–23. doi: 10.1270/jsbbs.23040 (PMC11442108; doi:10.1270/jsbbs.23040)
Supplement: Supplementary file 2 — Supplemental Tables [file 74_114_s2.pdf]

doi: 10.1270/jsbbs.23040

Supplemental Table 1. Planting methods and cropping seasons in yield trials in the NARO rice historical phenotype dataset

Research station	Transplanting			Direct seeding[a]		Cropping season[b]			
	Early	Standard	Late	Flooded	Drained	1	2	3	4
HARC	0	524	0	119	4	0	0	524	0
TARC	0	607	4	177	11	0	0	607	4
CARC	0	1082	38	96	0	0	0	1082	38
NICS	1225	0	153	211	50	0	1225	0	153
WARC	103	611	0	41	112	103	0	611	0
KARC	79	611	4	149	10	0	79	611	4
Total	1407	3435	199	793	187	103	1304	3435	199
Percentage	23%	57%	3%	13%	3%				

[a] Direct seeding in flooded or well-drained paddy field

[b] 1: early, 2: mid-early, 3: mid-late, and 4: late

Supplemental Table 2. Planting density in yield trials in the NARO rice historical phenotype dataset

	Transplanting (hills/m^2)				Direct seeding (g/m^2)			
	Min	Max	Mode	Mean	Min	Max	Mode	Mean
HARC	13.3	26.7	24.0	24.0	6.0	10.0	10.0	8.5
TARC	11.1	22.4	22.2	22.2	4.4	12.0	5.0	5.6
CARC	11.1	18.5	18.5	18.5	4.0	5.0	5.0	4.1
NICS	22.2	22.2	22.2	22.2	3.3	6.7	4.0	4.3
WARC	18.5	22.2	20.8	20.7	3.0	8.9	3.0	4.1
KARC	20.8	23.8	20.8	21.1	3.5	5.0	5.0	4.4

Supplemental Table 3. Nitrogen application in yield trials in the NARO rice historical phenotype dataset

	Basal dressing (g/m^2)				Topdressing (g/m^2)				Total (g/m^2)			
	Min	Max	Mode	Mean	Min	Max	Mode	Mean	Min	Max	Mode	Mean
HARC	4	17	10	9	0	5	0	0	4	19	10	9
TARC	4	11	7	7	0	9	2	3	5	17	7	10
CARC	3	8	4	5	0	23	2	3	4	29	6	7
NICS	4	24	8	9	0	4	0	0	4	24	8	10
WARC	3	18	6	7	0	13	1	2	6	20	7	9
KARC	4	18	5	8	0	11	0	3	8	18	8	10

Supplemental Table 4. Seeding and transplanting dates in yield trials in the NARO rice historical phenotype dataset

	Transplanting time or field condition for direct seeding	Seeding date				Transplanting date			
		Earliest	Latest	Mode	Mean	Earliest	Latest	Mode	Mean
HARC	Standard transplanting (Cropping season 3)	13-Apr	20-Apr	18-Apr	16-Apr	18-May	23-May	22-May	20-May
	Direct seeding in flooded paddy field	13-Apr	16-May	18-Apr	21-Apr	-	-	-	-
	Direct seeding in well-drained paddy field	15-May	15-May	15-May	15-May	-	-	-	-
TARC	Standard transplanting (Cropping season 3)	12-Apr	06-May	23-Apr	21-Apr	16-May	29-May	18-May	20-May
	Late transplanting (Cropping season 4)	14-May	14-May	14-May	14-May	14-Jun	14-Jun	14-Jun	14-Jun
	Direct seeding in flooded paddy field	12-Apr	16-May	23-Apr	26-Apr	-	-	-	-
	Direct seeding in well-drained paddy field	5-Apr	30-May	19-Apr	17-Apr	-	-	-	-
CARC	Standard transplanting (Cropping season 3)	05-Apr	21-Apr	16-Apr	15-Apr	10-May	21-May	15-May	16-May
	Late transplanting (Cropping season 4)	23-May	30-May	30-May	26-May	17-Jun	22-Jun	22-Jun	19-Jun
	Direct seeding in flooded paddy field	8-May	13-May	8-May	10-May	-	-	-	-
NICS	Early transplanting (Cropping season 2)	14-Apr	24-Apr	22-Apr	19-Apr	13-May	19-May	16-May	16-May
	Late transplanting (Cropping season 4)	29-May	14-Jun	04-Jun	06-Jun	21-Jun	28-Jun	24-Jun	24-Jun
	Direct seeding in flooded paddy field	14-Apr	22-Jun	22-Apr	27-Apr	-	-	-	-
	Direct seeding in well-drained paddy field	24-Apr	22-Jun	19-Jun	20-May	-	-	-	-
WARC	Early transplanting (Cropping season 1)	03-Apr	12-Apr	05-Apr	07-Apr	07-May	14-May	14-May	10-May
	Standard transplanting (Cropping season 3)	01-May	13-May	06-May	07-May	01-Jun	13-Jun	08-Jun	06-Jun
	Direct seeding in flooded paddy field	3-Apr	28-May	6-May	3-May	-	-	-	-
	Direct seeding in well-drained paddy field	12-Apr	27-Jun	23-Apr	28-Apr	-	-	-	-
KARC	Early transplanting (Cropping season 2)	19-Apr	01-May	20-Apr	23-Apr	17-May	24-May	19-May	19-May
	Standard transplanting (Cropping season 3)	18-May	06-Jun	25-May	22-May	13-Jun	25-Jun	19-Jun	17-Jun
	Late transplanting (Cropping season 4)	09-Jul	09-Jul	09-Jul	09-Jul	21-Jul	21-Jul	21-Jul	21-Jul
	Direct seeding in flooded paddy field	19-Apr	9-Jul	25-May	23-May	-	-	-	-
	Direct seeding in well-drained paddy field	4-Jun	4-Jun	4-Jun	4-Jun	-	-	-	-

Supplemental Table 5. The characteristics of the varieties analyzed in the NARO Rice Historical Phenotype Dataset.

Variety	Release Year	Number of data	Years for experiments [a]	Locations for experiments [a]	Amount of manure [a]	Number of data in each trait: Days to heading	Days to maturity	Ripening period	Culm length	Panicle length	Number of panicles	Number of grains per panicle	Plant weight	Grain yield	Harvest index	Thousand-grain weight	Grain quality	Lodging degree	Amylose content	Protein content
NARO-HIST001	1972	8	1995(1);1996(3);	CARC(7);NICS(1);	0-0.5(0);0.5-1.	8	8	8	8	8	8	8	8	8	8	8	8	8	3	4
NARO-HIST002	1977	8	1994(2);1995(1);	KARC(8);	0-0.5(0);0.5-1.	7	7	7	7	7	7	7	7	7	7	7	7	7	3	3
NARO-HIST003	1982	82	1997(2);1998(7);	CARC(5);NICS(77);	0-0.5(0);0.5-1.	82	82	82	82	82	82	82	82	82	82	82	82	82	4	3
NARO-HIST004	1983	78	1994(1);1995(3);	NICS(72);WARC(6);	0-0.5(0);0.5-1.	76	75	75	76	76	76	76	75	76	75	76	76	76	7	6
NARO-HIST005	1986	1	2001(1);	NICS(1);	0-0.5(0);0.5-1.	1	1	1	1	1	1	1	1	1	1	1	1	1	0	0
NARO-HIST006	1990	21	1998(1);1999(1);	CARC(8);WARC(13);	0-0.5(0);0.5-1.	21	21	21	21	21	21	21	21	21	21	21	21	21	1	2
NARO-HIST007	1992	19	2012(4);2013(7);	CARC(1);NICS(18);	0-0.5(0);0.5-1.	19	19	19	19	19	19	19	19	19	19	19	19	19	0	0
NARO-HIST008	2001	4	2009(2);2010(1);	NICS(3);WARC(1);	0-0.5(0);0.5-1.	4	4	4	4	4	4	4	4	4	4	4	4	4	0	0
NARO-HIST009	1961	334	1991(1);1992(1);	CARC(80);KARC(47);I	0-0.5(1);0.5-1.	331	320	320	331	331	331	321	311	323	304	322	322	331	48	40
NARO-HIST010	1950	8	1992(1);1993(1);	WARC(8);	0-0.5(0);0.5-1.	8	8	8	8	8	8	8	8	8	8	8	8	8	0	0
NARO-HIST011	1982	156	1992(1);1994(3);	CARC(58);NICS(23);T	0-0.5(1);0.5-1.	156	154	154	156	156	156	152	107	153	107	153	155	156	28	28
NARO-HIST012	1989	2	2015(1);2016(1);	TARC(2);	0-0.5(0);0.5-1.	2	2	2	2	2	2	2	2	2	2	2	2	2	0	0
NARO-HIST013	1980	5	1996(2);1997(1);	CARC(5);	0-0.5(0);0.5-1.	5	5	5	5	5	5	5	5	5	5	5	5	5	2	3
NARO-HIST014	1995	2	2000(1);2007(1);	TARC(2);	0-0.5(0);0.5-1.	2	2	2	2	2	2	2	0	2	0	2	2	2	0	0
NARO-HIST015	1999	2	2010(1);2011(1);	TARC(2);	0-0.5(0);0.5-1.	2	2	2	2	2	2	2	0	2	0	2	2	2	1	2
NARO-HIST016	1994	4	2009(2);2010(2);	NICS(4);	0-0.5(0);0.5-1.	4	4	4	4	4	4	4	4	4	4	4	4	4	0	0
NARO-HIST017	2005	2	2011(1);2012(1);	KARC(2);	0-0.5(0);0.5-1.	2	2	2	2	2	2	2	2	2	2	2	2	2	1	1
NARO-HIST018	1973	10	1994(2);1995(1);	KARC(10);	0-0.5(0);0.5-1.	9	9	9	9	9	9	9	8	9	8	9	9	9	3	3
NARO-HIST019	1977	3	1994(1);1995(1);	WARC(3);	0-0.5(0);0.5-1.	3	3	3	3	3	3	3	3	3	3	3	3	3	0	0
NARO-HIST020	1993	2	2004(1);2005(1);	KARC(2);	0-0.5(0);0.5-1.	2	2	2	2	2	2	2	2	2	2	2	2	2	0	0
NARO-HIST021	1981	14	1993(1);1994(2);	KARC(6);WARC(8);	0-0.5(0);0.5-1.	14	14	14	14	14	14	14	14	14	14	14	13	14	0	0
NARO-HIST022	1982	7	1993(1);1994(2);	WARC(7);	0-0.5(0);0.5-1.	7	7	7	7	7	7	7	7	7	7	7	6	7	0	0
NARO-HIST023	1984	14	1998(1);1999(3);	NICS(4);WARC(10);	0-0.5(0);0.5-1.	14	12	12	14	14	14	8	13	8	8	11	10	14	1	0
NARO-HIST024	1993	3	1996(1);1997(1);	WARC(3);	0-0.5(0);0.5-1.	3	3	3	3	3	3	2	3	2	2	3	2	3	0	0
NARO-HIST025	1995	44	1999(1);2000(1);	CARC(3);KARC(17);N	0-0.5(0);0.5-1.	44	35	35	44	44	44	28	37	28	27	31	28	43	3	7
NARO-HIST026	1995	20	1999(1);2000(2);	KARC(10);NICS(6);W	0-0.5(0);0.5-1.	20	16	16	19	19	19	6	17	6	6	12	10	20	0	0
NARO-HIST027	1996	1	1995(1);	WARC(1);	0-0.5(0);0.5-1.	1	1	1	1	1	1	1	1	1	1	1	1	1	0	0
NARO-HIST028	1996	4	1992(1);1993(1);	WARC(4);	0-0.5(0);0.5-1.	4	4	4	4	4	4	4	4	4	4	4	4	4	0	0
NARO-HIST029	1996	3	1993(1);1994(1);	WARC(3);	0-0.5(0);0.5-1.	3	3	3	3	3	3	3	3	3	3	3	3	3	0	0
NARO-HIST030	1996	2	1994(1);1995(1);	WARC(2);	0-0.5(0);0.5-1.	2	2	2	2	2	2	2	2	2	2	2	2	2	0	0
NARO-HIST031	1996	3	1993(1);1994(1);	WARC(3);	0-0.5(0);0.5-1.	3	3	3	3	3	3	3	3	3	3	3	2	3	0	0
NARO-HIST032	1996	3	1993(1);1994(1);	WARC(3);	0-0.5(0);0.5-1.	3	3	3	3	3	3	3	3	3	3	3	3	3	0	0
NARO-HIST033	1996	3	1993(1);1994(1);	WARC(3);	0-0.5(0);0.5-1.	3	3	3	3	3	3	3	3	3	3	3	3	3	0	0
NARO-HIST034	1997	1	1995(1);	NICS(1);	0-0.5(0);0.5-1.	1	1	1	1	1	1	1	0	1	0	1	1	1	0	0
NARO-HIST035	1997	1	1996(1);	NICS(1);	0-0.5(0);0.5-1.	1	1	1	1	1	1	1	0	1	0	1	1	1	0	0
NARO-HIST036	1997	3	1994(1);1995(1);	WARC(3);	0-0.5(0);0.5-1.	3	3	3	3	3	3	3	3	3	3	3	3	3	0	0
NARO-HIST037	1997	4	1993(1);1994(1);	WARC(4);	0-0.5(0);0.5-1.	4	4	4	4	4	4	4	4	4	4	4	4	4	0	0
NARO-HIST038	1997	2	1995(1);1996(1);	WARC(2);	0-0.5(0);0.5-1.	2	2	2	2	2	2	2	2	2	2	2	0	2	0	0
NARO-HIST039	1997	1	1996(1);	WARC(1);	0-0.5(0);0.5-1.	1	1	1	1	1	1	1	1	1	1	1	1	1	0	0
NARO-HIST040	1997	1	1996(1);	WARC(1);	0-0.5(0);0.5-1.	1	1	1	1	1	1	1	1	1	1	1	1	1	0	0
NARO-HIST041	1997	1	1996(1);	WARC(1);	0-0.5(0);0.5-1.	1	1	1	1	1	1	1	1	1	1	1	1	1	0	0
NARO-HIST042	1998	2	1996(1);1997(1);	WARC(2);	0-0.5(0);0.5-1.	2	2	2	2	2	2	2	2	2	2	2	2	2	0	0
NARO-HIST043	1998	3	1995(1);1996(1);	WARC(3);	0-0.5(0);0.5-1.	3	3	3	3	3	3	3	3	3	3	3	3	3	0	0
NARO-HIST044	1998	2	1996(1);1997(1);	WARC(2);	0-0.5(0);0.5-1.	2	2	2	2	2	2	2	2	2	2	2	1	2	0	0
NARO-HIST045	1999	3	1996(1);1997(1);	WARC(3);	0-0.5(0);0.5-1.	3	3	3	3	3	3	3	3	3	3	3	3	3	0	0
NARO-HIST046	1999	4	1995(1);1996(1);	WARC(4);	0-0.5(0);0.5-1.	4	4	4	4	4	4	4	4	4	4	4	4	4	0	0
NARO-HIST047	2000	7	1995(1);1996(2);	NICS(2);WARC(5);	0-0.5(0);0.5-1.	4	4	4	4	4	4	2	2	4	2	2	7	4	0	0
NARO-HIST048	2000	4	1996(1);1997(1);	WARC(4);	0-0.5(0);0.5-1.	4	4	4	4	4	4	4	4	4	4	4	4	4	0	0
NARO-HIST049	2000	9	1998(1);1999(1);	NICS(2);WARC(7);	0-0.5(1);0.5-1.	9	9	9	9	9	9	9	9	9	9	9	9	9	0	0
NARO-HIST050	2001	3	1998(1);1999(1);	WARC(3);	0-0.5(0);0.5-1.	3	3	3	3	3	3	3	3	3	3	3	3	3	0	0
NARO-HIST051	2001	4	1997(1);1998(1);	WARC(4);	0-0.5(0);0.5-1.	4	4	4	4	4	4	4	4	4	4	4	4	4	0	0
NARO-HIST052	2002	4	1998(1);1999(1);	WARC(4);	0-0.5(0);0.5-1.	4	4	4	4	4	4	4	4	4	4	4	4	4	0	0
NARO-HIST053	2002	3	1999(1);2000(1);	WARC(3);	0-0.5(0);0.5-1.	3	2	2	3	3	3	3	3	3	3	3	3	3	0	0
NARO-HIST054	2002	5	1998(1);1999(1);	WARC(5);	0-0.5(0);0.5-1.	5	4	4	5	5	5	4	4	4	4	4	4	5	0	0
NARO-HIST055	2003	3	2000(1);2001(1);	WARC(3);	0-0.5(0);0.5-1.	3	3	3	3	3	3	3	3	3	3	3	3	3	0	0
NARO-HIST056	2003	2	2001(1);2002(1);	WARC(2);	0-0.5(0);0.5-1.	2	2	2	2	2	2	2	2	2	2	2	2	2	0	0
NARO-HIST057	2003	3	2002(1);2008(1);	KARC(1);WARC(2);	0-0.5(0);0.5-1.	3	3	3	3	3	3	3	3	3	3	3	3	3	1	0
NARO-HIST058	2004	2	2002(1);2003(1);	WARC(2);	0-0.5(0);0.5-1.	2	2	2	2	2	2	2	2	2	2	2	2	2	0	0
NARO-HIST059	2004	4	2000(1);2001(1);	WARC(4);	0-0.5(0);0.5-1.	4	4	4	4	4	4	4	4	4	4	4	4	4	0	0
NARO-HIST060	2005	1	2001(1);	WARC(1);	0-0.5(0);0.5-1.	1	1	1	1	1	1	1	1	1	1	1	1	1	0	0
NARO-HIST061	2005	2	2003(1);2004(1);	WARC(2);	0-0.5(0);0.5-1.	2	2	2	2	2	2	2	2	2	2	2	2	2	0	0
NARO-HIST062	2005	1	2004(1);	WARC(1);	0-0.5(0);0.5-1.	1	1	1	1	1	1	1	1	1	1	1	1	1	0	0
NARO-HIST063	2005	4	2001(1);2002(1);	WARC(4);	0-0.5(0);0.5-1.	4	4	4	4	4	4	4	4	4	4	4	4	4	0	0
NARO-HIST064	2006	5	2004(1);2005(1);	WARC(5);	0-0.5(0);0.5-1.	5	5	5	5	5	5	5	5	5	5	5	5	5	2	0
NARO-HIST065	2007	3	2005(1);2006(2);	NICS(1);WARC(2);	0-0.5(0);0.5-1.	3	3	3	3	3	3	3	2	3	2	3	3	3	0	0
NARO-HIST066	2007	3	2004(1);2005(1);	WARC(3);	0-0.5(0);0.5-1.	3	3	3	3	3	3	3	3	3	3	3	3	3	0	0
NARO-HIST067	2008	2	2006(1);2007(1);	WARC(2);	0-0.5(0);0.5-1.	2	2	2	2	2	2	2	2	2	2	2	2	2	0	0
NARO-HIST068	2008	3	2005(1);2006(1);	WARC(3);	0-0.5(0);0.5-1.	3	3	3	3	3	3	3	3	3	3	3	3	3	0	0
NARO-HIST069	2008	3	2005(1);2006(1);	WARC(3);	0-0.5(0);0.5-1.	3	3	3	3	3	3	3	3	3	3	3	3	3	0	0
NARO-HIST070	2008	3	2007(1);2013(1);	WARC(3);	0-0.5(0);0.5-1.	3	1	1	3	2	2	0	0	0	0	2	1	3	0	0
NARO-HIST071	2009	2	2007(1);2008(1);	WARC(2);	0-0.5(0);0.5-1.	2	2	2	2	2	2	2	2	2	2	2	2	2	0	0
NARO-HIST072	2009	8	2007(1);2008(1);	KARC(1);WARC(7);	0-0.5(0);0.5-1.	8	8	8	8	8	8	8	7	8	7	8	8	8	2	2
NARO-HIST073	2009	1	2008(1);	WARC(1);	0-0.5(0);0.5-1.	1	1	1	1	1	1	1	1	1	1	1	1	1	1	0
NARO-HIST074	2010	4	2007(1);2008(1);	CARC(1);WARC(3);	0-0.5(0);0.5-1.	4	4	4	4	4	4	4	3	4	3	4	4	4	0	0
NARO-HIST075	2010	5	2007(1);2008(1);	CARC(1);NICS(2);WA	0-0.5(0);0.5-1.	5	5	5	5	5	5	5	2	5	2	5	5	5	0	0
NARO-HIST076	2010	6	2008(1);2009(2);	CARC(2);TARC(1);WA	0-0.5(0);0.5-1.	6	2	2	6	6	6	1	1	1	1	2	2	6	0	0
NARO-HIST077	2011	7	2007(1);2008(1);	CARC(2);NICS(1);WA	0-0.5(0);0.5-1.	7	7	7	7	7	7	7	7	7	7	7	7	7	0	0
NARO-HIST078	2011	2	2009(1);2010(1);	WARC(2);	0-0.5(0);0.5-1.	2	2	2	2	2	2	2	2	2	2	2	2	2	0	0
NARO-HIST079	2011	2	2009(1);2010(1);	WARC(2);	0-0.5(0);0.5-1.	2	2	2	2	2	2	2	2	2	2	2	2	2	0	0
NARO-HIST080	2011	4	2010(1);2011(1);	WARC(4);	0-0.5(0);0.5-1.	4	4	4	4	4	4	4	4	4	4	4	4	4	1	1
NARO-HIST081	2011	3	2010(1);2011(2);	NICS(1);WARC(2);	0-0.5(0);0.5-1.	3	3	3	3	3	3	3	2	3	2	3	3	3	0	0
NARO-HIST082	2013	3	2011(1);2012(2);	NICS(1);WARC(2);	0-0.5(0);0.5-1.	3	3	3	3	3	3	3	3	3	3	3	3	3	0	0
NARO-HIST083	2013	3	2011(1);2012(2);	NICS(1);WARC(2);	0-0.5(0);0.5-1.	3	3	3	3	3	3	3	3	3	3	3	3	3	1	0
NARO-HIST084	2013	3	2011(1);2012(2);	KARC(1);WARC(2);	0-0.5(0);0.5-1.	3	3	3	3	3	3	3	3	3	3	3	3	3	0	0
NARO-HIST085	2013	3	2011(1);2012(2);	KARC(1);WARC(2);	0-0.5(0);0.5-1.	3	3	3	3	3	3	3	3	3	3	3	3	3	0	0
NARO-HIST086	2013	2	2011(1);2012(1);	WARC(2);	0-0.5(0);0.5-1.	2	1	1	2	2	2	0	2	0	0	2	2	2	1	0
NARO-HIST087	2014	4	2011(1);2012(2);	KARC(1);WARC(3);	0-0.5(0);0.5-1.	4	4	4	4	4	4	4	4	4	4	4	4	4	1	0
NARO-HIST088	2014	8	2011(1);2012(2);	KARC(1);NICS(1);WA	0-0.5(0);0.5-1.	8	8	8	8	8	8	8	8	8	8	8	8	7	0	0
NARO-HIST089	2015	3	2013(1);2014(2);	KARC(1);WARC(2);	0-0.5(0);0.5-1.	3	3	3	3	3	3	3	3	3	3	3	3	3	1	1
NARO-HIST090	2015	2	2013(1);2014(1);	WARC(2);	0-0.5(0);0.5-1.	2	0	0	2	1	1	0	0	0	0	1	0	2	0	0
NARO-HIST091	2015	4	2013(1);2014(1);	WARC(4);	0-0.5(0);0.5-1.	4	2	2	4	3	3	2	2	2	2	3	2	4	1	1
NARO-HIST092	2016	2	2014(1);2015(1);	WARC(2);	0-0.5(0);0.5-1.	2	2	2	2	2	2	2	2	2	2	2	2	2	1	1
NARO-HIST093	2016	6	2014(1);2015(3);	CARC(1);NICS(1);WA	0-0.5(0);0.5-1.	6	6	6	6	6	6	4	6	4	4	6	6	6	0	0
NARO-HIST094	2016	4	2014(1);2015(2);	KARC(1);WARC(3);	0-0.5(0);0.5-1.	4	4	4	4	4	4	4	3	4	3	4	4	4	1	1
NARO-HIST095	2016	1	2016(1);	WARC(1);	0-0.5(0);0.5-1.	1	0	0	1	1	1	0	0	0	0	0	0	1	0	0
NARO-HIST096	2016	1	2016(1);	WARC(1);	0-0.5(0);0.5-1.	1	0	0	1	1	1	0	0	0	0	0	0	1	0	0
NARO-HIST097	2018	3	2016(1);2017(2);	CARC(1);WARC(2);	0-0.5(0);0.5-1.	3	3	3	3	3	3	3	2	3	2	3	3	3	1	1
NARO-HIST098	2019	2	2017(1);2018(1);	WARC(2);	0-0.5(0);0.5-1.	2	2	2	2	2	2	2	2	2	2	2	2	2	1	1
NARO-HIST099	2019	2	2017(1);2018(1);	WARC(2);	0-0.5(0);0.5-1.	2	2	2	2	2	2	2	2	2	2	2	2	2	1	1
NARO-HIST100	2019	2	2017(1);2018(1);	WARC(2);	0-0.5(0);0.5-1.	2	2	2	2	2	2	2	2	2	2	2	2	2	0	0
NARO-HIST101	1953	155	1994(2);1995(5);	CARC(82);KARC(1);N	0-0.5(1);0.5-1.	145	144	144	145	145	145	143	140	144	140	144	147	145	34	29
NARO-HIST102	1988	31	1995(1);1996(2);	CARC(31);	0-0.5(0);0.5-1.	31	31	31	31	31	31	31	31	31	31	31	31	31	10	12
NARO-HIST103	2005	21	2010(1);2011(2);	CARC(12);KARC(2);W	0-0.5(0);0.5-1.	21	20	20	21	21	21	21	20	21	20	21	21	21	1	1
NARO-HIST104	1974	37	1992(1);1994(2);	CARC(21);NICS(1);TA	0-0.5(0);0.5-1.	37	34	34	37	37	37	32	21	32	21	35	35	37	4	5
NARO-HIST105	1989	2	2006(1);2007(1);	NICS(2);	0-0.5(0);0.5-1.	2	2	2	2	2	2	2	2	2	2	2	2	2	0	0
NARO-HIST106	1990	1	2000(1);	TARC(1);	0-0.5(0);0.5-1.	1	1	1	1	1	1	1	0	1	0	1	1	1	0	0

NARO-HIST107	2004	1	2011(1);	TARC(1);	0-0.5(0);0.5-1.	1	1	1	1	1	1	0	0	0	0	1	1	1	0	0
NARO-HIST108	1949	17	1995(1);1996(1);	WARC(17);	0-0.5(0);0.5-1.	16	16	16	17	17	17	17	17	17	17	17	17	17	0	0
NARO-HIST109	1989	2	2001(1);2002(1);	HARC(2);	0-0.5(0);0.5-1.	2	2	2	2	2	2	2	1	2	1	2	2	2	1	1
NARO-HIST110	1993	20	1997(1);1999(2);	HARC(20);	0-0.5(0);0.5-1.	14	11	11	14	14	14	4	11	4	4	8	6	14	5	10
NARO-HIST111	1994	3	1998(2);1999(1);	HARC(3);	0-0.5(0);0.5-1.	3	3	3	3	3	3	3	3	3	3	3	3	3	3	2
NARO-HIST112	1997	1	1996(1);	HARC(1);	0-0.5(0);0.5-1.	1	1	1	1	1	1	1	1	1	1	1	1	1	0	0
NARO-HIST113	1998	6	1996(1);1997(1);	HARC(6);	0-0.5(0);0.5-1.	6	6	6	6	6	6	5	6	5	5	6	6	6	4	4
NARO-HIST114	1999	2	1997(1);1998(1);	HARC(2);	0-0.5(0);0.5-1.	2	2	2	2	2	2	2	2	2	2	2	2	2	1	1
NARO-HIST115	1999	2	1997(1);1998(1);	HARC(2);	0-0.5(0);0.5-1.	2	2	2	2	2	2	2	2	2	2	2	2	2	0	1
NARO-HIST116	2000	2	1998(1);1999(1);	HARC(2);	0-0.5(0);0.5-1.	2	2	2	2	2	2	2	2	2	2	2	2	2	2	2
NARO-HIST117	2000	13	1998(1);1999(1);	HARC(13);	0-0.5(0);0.5-1.	8	8	8	8	8	8	8	8	8	8	8	8	8	10	13
NARO-HIST118	2001	4	1999(1);2000(1);	HARC(4);	0-0.5(0);0.5-1.	4	4	4	4	4	4	4	4	4	4	4	4	4	2	4
NARO-HIST119	2002	3	1999(1);2000(1);	HARC(3);	0-0.5(0);0.5-1.	3	3	3	3	3	3	3	3	3	3	3	3	3	1	1
NARO-HIST120	2002	2	2000(1);2001(1);	HARC(2);	0-0.5(0);0.5-1.	2	2	2	2	2	2	2	2	2	2	2	2	2	0	1
NARO-HIST121	2002	2	2000(1);2001(1);	HARC(2);	0-0.5(0);0.5-1.	2	2	2	2	2	2	2	2	2	2	2	2	2	0	1
NARO-HIST122	2003	2	2001(1);2002(1);	HARC(2);	0-0.5(0);0.5-1.	2	2	2	2	2	2	2	2	2	2	2	2	2	1	1
NARO-HIST123	2003	2	2001(1);2002(1);	HARC(2);	0-0.5(0);0.5-1.	2	2	2	2	2	2	2	1	2	1	2	2	2	1	1
NARO-HIST124	2004	6	2002(1);2003(1);	HARC(6);	0-0.5(0);0.5-1.	6	6	6	6	6	6	6	6	6	6	6	2	6	6	6
NARO-HIST125	2004	2	2002(1);2003(1);	HARC(2);	0-0.5(0);0.5-1.	2	2	2	2	2	2	0	2	0	0	1	2	2	0	0
NARO-HIST126	2005	10	2003(1);2004(2);	HARC(10);	0-0.5(0);0.5-1.	10	10	10	10	10	10	10	10	10	10	10	10	10	7	9
NARO-HIST127	2005	5	2003(1);2004(2);	HARC(5);	0-0.5(0);0.5-1.	5	5	5	5	5	5	5	5	5	5	5	5	5	4	4
NARO-HIST128	2005	4	2003(1);2004(3);	HARC(4);	0-0.5(0);0.5-1.	4	1	1	4	4	4	0	1	0	0	0	1	4	0	0
NARO-HIST129	2006	3	2004(1);2005(2);	HARC(3);	0-0.5(0);0.5-1.	3	3	3	3	3	3	3	3	3	3	3	3	3	1	3
NARO-HIST130	2006	3	2004(1);2005(2);	HARC(3);	0-0.5(0);0.5-1.	3	3	3	3	3	3	2	3	3	3	2	3	3	0	3
NARO-HIST131	2006	3	2004(1);2005(2);	HARC(3);	0-0.5(0);0.5-1.	3	3	3	3	3	3	2	3	3	3	2	3	3	0	3
NARO-HIST132	2006	25	2003(1);2004(3);	HARC(25);	0-0.5(3);0.5-1.	25	21	21	25	25	25	11	21	11	11	19	19	25	7	7
NARO-HIST133	2007	3	2005(1);2006(2);	HARC(3);	0-0.5(0);0.5-1.	3	3	3	3	3	3	3	3	3	3	3	3	3	3	3
NARO-HIST134	2008	18	2006(1);2007(3);	HARC(18);	0-0.5(3);0.5-1.	18	16	16	18	18	18	9	17	9	9	15	15	18	7	7
NARO-HIST135	2009	5	2006(1);2007(2);	HARC(5);	0-0.5(0);0.5-1.	5	5	5	5	5	5	5	5	5	5	5	5	5	5	5
NARO-HIST136	2009	6	2007(1);2008(5);	HARC(6);	0-0.5(0);0.5-1.	6	6	6	6	6	6	0	6	0	0	6	6	6	1	1
NARO-HIST137	2010	4	2007(1);2008(1);	HARC(4);	0-0.5(0);0.5-1.	4	4	4	4	4	4	4	4	4	4	4	3	4	4	4
NARO-HIST138	2010	3	2008(1);2009(2);	HARC(3);	0-0.5(0);0.5-1.	3	3	3	3	3	3	3	3	3	3	3	3	3	3	3
NARO-HIST139	2010	2	2009(2);	HARC(2);	0-0.5(0);0.5-1.	2	2	2	2	2	2	2	2	2	2	2	2	2	2	2
NARO-HIST140	2010	3	2008(1);2009(2);	HARC(3);	0-0.5(0);0.5-1.	3	3	3	3	3	3	0	3	0	0	3	3	3	0	0
NARO-HIST141	2010	4	2007(1);2008(1);	HARC(4);	0-0.5(0);0.5-1.	4	2	2	4	4	4	0	4	0	0	4	3	4	1	1
NARO-HIST142	2012	5	2009(1);2010(2);	HARC(5);	0-0.5(0);0.5-1.	5	5	5	5	5	5	5	5	5	5	5	5	5	5	5
NARO-HIST143	2012	5	2010(2);2011(3);	HARC(5);	0-0.5(1);0.5-1.	5	5	5	5	5	5	5	5	5	5	5	5	5	0	0
NARO-HIST144	2012	12	2007(1);2008(2);	HARC(12);	0-0.5(3);0.5-1.	12	12	12	11	11	11	11	11	11	11	11	9	12	6	6
NARO-HIST145	2013	3	2011(1);2012(2);	HARC(3);	0-0.5(0);0.5-1.	3	3	3	3	3	3	3	3	3	3	3	3	3	2	2
NARO-HIST146	2013	3	2011(1);2012(2);	HARC(3);	0-0.5(0);0.5-1.	3	3	3	3	3	3	3	3	3	3	3	3	3	3	3
NARO-HIST147	2014	4	2011(1);2012(2);	HARC(4);	0-0.5(0);0.5-1.	4	4	4	3	3	3	3	4	4	4	4	4	4	4	4
NARO-HIST148	2014	4	2011(1);2012(2);	HARC(4);	0-0.5(0);0.5-1.	4	4	4	4	4	4	4	4	4	4	4	0	4	4	4
NARO-HIST149	2014	2	2012(1);2013(1);	HARC(2);	0-0.5(0);0.5-1.	2	2	2	2	2	2	2	2	2	2	2	2	2	1	1
NARO-HIST150	2014	3	2012(1);2013(2);	HARC(3);	0-0.5(0);0.5-1.	3	3	3	3	3	3	0	3	0	0	3	3	3	1	1
NARO-HIST151	2014	3	2012(1);2013(2);	HARC(3);	0-0.5(0);0.5-1.	3	3	3	3	3	3	0	3	0	0	2	2	3	1	1
NARO-HIST152	2014	4	2012(1);2013(3);	HARC(4);	0-0.5(0);0.5-1.	4	3	3	4	4	4	0	3	0	0	2	2	4	1	1
NARO-HIST153	2014	4	2012(1);2013(3);	HARC(4);	0-0.5(0);0.5-1.	4	3	3	4	4	4	0	3	0	0	2	2	4	1	1
NARO-HIST154	2015	2	2013(1);2014(1);	HARC(2);	0-0.5(0);0.5-1.	2	2	2	2	2	2	2	2	2	2	2	2	2	2	2
NARO-HIST155	2015	2	2013(1);2014(1);	HARC(2);	0-0.5(0);0.5-1.	2	2	2	2	2	2	2	2	2	2	2	2	2	2	2
NARO-HIST156	2016	2	2014(1);2015(1);	HARC(2);	0-0.5(0);0.5-1.	2	2	2	2	2	2	2	2	2	2	2	2	2	2	2
NARO-HIST157	2017	3	2015(1);2016(2);	HARC(3);	0-0.5(0);0.5-1.	3	3	3	3	3	3	3	3	3	3	3	3	3	2	3
NARO-HIST158	2018	2	2016(1);2017(1);	HARC(2);	0-0.5(0);0.5-1.	2	2	2	2	2	2	2	2	2	2	2	2	2	1	2
NARO-HIST159	2018	2	2016(1);2017(1);	HARC(2);	0-0.5(0);0.5-1.	2	2	2	2	2	2	2	2	2	2	2	2	2	1	2
NARO-HIST160	2018	3	2015(1);2016(1);	HARC(3);	0-0.5(0);0.5-1.	3	3	3	3	3	3	3	3	3	3	3	3	3	3	3
NARO-HIST161	2019	2	2017(1);2018(1);	HARC(2);	0-0.5(0);0.5-1.	2	2	2	2	2	2	2	2	2	2	2	2	2	2	2
NARO-HIST162	1987	5	2000(1);2001(1);	HARC(5);	0-0.5(0);0.5-1.	3	3	3	3	3	3	0	3	0	0	3	3	3	0	2
NARO-HIST163	2001	1	2000(1);	HARC(1);	0-0.5(0);0.5-1.	1	1	1	1	1	1	1	1	1	1	1	1	1	0	0
NARO-HIST164	2003	11	1997(1);1998(1);	HARC(11);	0-0.5(0);0.5-1.	10	10	10	10	10	10	10	10	10	10	10	10	10	2	9
NARO-HIST165	1965	27	1994(1);1995(5);	CARC(24);TARC(3);	0-0.5(0);0.5-1.	26	26	26	26	26	26	26	26	26	26	26	26	25	13	15
NARO-HIST166	1969	25	1991(1);1992(1);	CARC(18);NICS(7);	0-0.5(0);0.5-1.	24	23	23	24	24	24	24	23	24	23	24	24	24	5	6
NARO-HIST167	1983	75	1994(1);1995(4);	CARC(42);KARC(7);N	0-0.5(0);0.5-1.	69	68	68	69	69	69	69	68	69	68	69	71	69	21	22
NARO-HIST168	1983	7	1994(1);1995(1);	CARC(7);	0-0.5(0);0.5-1.	7	7	7	7	7	7	7	7	7	7	7	7	7	2	2
NARO-HIST169	1984	1	2010(1);	CARC(1);	0-0.5(0);0.5-1.	1	1	1	1	1	1	1	1	1	1	1	1	1	0	0
NARO-HIST170	1987	5	2001(1);2002(2);	CARC(5);	0-0.5(0);0.5-1.	5	5	5	5	5	5	5	5	5	5	5	5	5	0	2
NARO-HIST171	1987	10	1997(2);1998(1);	CARC(10);	0-0.5(0);0.5-1.	10	8	8	10	10	10	8	8	8	8	8	9	10	1	1
NARO-HIST172	1990	30	1995(1);1996(4);	CARC(27);NICS(2);W	0-0.5(0);0.5-1.	27	27	27	27	27	27	27	27	27	27	27	27	27	12	12
NARO-HIST173	1990	2	2009(1);2010(1);	CARC(2);	0-0.5(0);0.5-1.	2	2	2	2	2	2	2	2	2	2	2	2	2	0	0
NARO-HIST174	1992	4	2007(1);2011(1);	CARC(4);	0-0.5(0);0.5-1.	4	4	4	4	4	4	4	4	4	4	4	4	4	4	1
NARO-HIST175	1993	2	2007(1);2008(1);	CARC(2);	0-0.5(0);0.5-1.	2	2	2	2	2	2	2	2	2	2	2	2	2	0	0
NARO-HIST176	1994	3	2006(1);2007(1);	CARC(3);	0-0.5(0);0.5-1.	3	3	3	3	3	3	3	3	3	3	3	3	3	0	0
NARO-HIST177	1994	5	2003(1);2004(1);	CARC(1);NICS(4);	0-0.5(0);0.5-1.	5	5	5	5	5	5	5	5	5	5	5	5	5	0	0
NARO-HIST178	1994	2	2008(1);2009(1);	CARC(2);	0-0.5(0);0.5-1.	2	2	2	2	2	2	2	2	2	2	2	2	2	0	0
NARO-HIST179	1996	2	1994(1);1995(1);	CARC(2);	0-0.5(0);0.5-1.	2	2	2	2	2	2	2	2	2	2	2	2	2	0	0
NARO-HIST180	1996	2	1994(1);1995(1);	CARC(2);	0-0.5(0);0.5-1.	2	2	2	2	2	2	2	2	2	2	2	2	2	0	0
NARO-HIST181	1997	3	1995(1);1996(2);	CARC(3);	0-0.5(0);0.5-1.	3	3	3	3	3	3	3	3	3	3	3	3	3	1	1
NARO-HIST182	1997	4	1995(1);1996(3);	CARC(3);TARC(1);	0-0.5(0);0.5-1.	4	4	4	4	4	4	4	4	4	4	4	4	4	2	2
NARO-HIST183	1997	3	1995(1);1996(2);	CARC(3);	0-0.5(0);0.5-1.	3	3	3	3	3	3	3	3	3	3	3	3	3	2	2
NARO-HIST184	1997	52	1994(1);1995(1);	CARC(46);NICS(6);	0-0.5(0);0.5-1.	49	49	49	49	49	49	49	48	49	48	49	49	49	9	5
NARO-HIST185	1997	11	1991(1);1992(1);	CARC(9);NICS(2);	0-0.5(0);0.5-1.	11	11	11	11	11	11	11	11	11	11	11	11	11	0	0
NARO-HIST186	1997	5	1993(1);1994(1);	CARC(5);	0-0.5(0);0.5-1.	5	5	5	5	5	5	5	5	5	5	5	5	5	0	0
NARO-HIST187	1998	3	1996(1);1997(2);	CARC(3);	0-0.5(0);0.5-1.	3	3	3	3	3	3	3	3	3	3	3	3	3	2	2
NARO-HIST188	1998	8	1994(2);1995(2);	CARC(6);TARC(2);	0-0.5(0);0.5-1.	8	8	8	8	8	8	8	7	8	7	8	8	7	0	0
NARO-HIST189	1998	3	1996(1);1997(2);	CARC(2);TARC(1);	0-0.5(0);0.5-1.	3	3	3	3	3	3	3	3	3	3	3	3	3	2	2
NARO-HIST190	1999	3	1997(1);1998(2);	CARC(3);	0-0.5(0);0.5-1.	3	3	3	3	3	3	3	3	3	3	3	3	3	1	2
NARO-HIST191	1999	4	1997(1);1998(3);	CARC(3);TARC(1);	0-0.5(0);0.5-1.	4	4	4	4	4	4	4	4	4	4	4	4	4	1	2
NARO-HIST192	1999	29	1997(1);1998(1);	CARC(14);KARC(2);N	0-0.5(0);0.5-1.	29	19	19	29	29	29	17	18	17	16	21	18	29	2	5
NARO-HIST193	1999	5	1996(1);1997(2);	CARC(3);TARC(2);	0-0.5(0);0.5-1.	5	5	5	5	5	5	5	5	5	5	5	5	5	1	2
NARO-HIST194	2000	3	1998(1);1999(2);	CARC(3);	0-0.5(0);0.5-1.	3	3	3	3	3	3	3	3	3	3	3	3	3	2	2
NARO-HIST195	2000	3	1998(1);1999(2);	CARC(3);	0-0.5(0);0.5-1.	3	3	3	3	3	3	3	3	3	3	3	3	3	2	2
NARO-HIST196	2001	3	1999(1);2000(2);	CARC(3);	0-0.5(0);0.5-1.	3	3	3	3	3	3	3	3	3	3	3	3	3	2	2
NARO-HIST197	2001	7	1996(1);1997(2);	CARC(7);	0-0.5(0);0.5-1.	7	7	7	7	7	7	7	7	7	7	7	7	7	0	0
NARO-HIST198	2001	59	1998(1);1999(1);	CARC(24);KARC(7);N	0-0.5(0);0.5-1.	59	57	57	59	59	59	56	52	56	51	57	57	58	7	7
NARO-HIST199	2002	5	1999(1);2000(2);	CARC(5);	0-0.5(0);0.5-1.	5	5	5	5	5	5	5	5	5	5	5	5	5	3	3
NARO-HIST200	2002	5	1999(1);2000(2);	CARC(5);	0-0.5(0);0.5-1.	5	5	5	5	5	5	5	5	5	5	5	5	5	3	3
NARO-HIST201	2002	3	2000(1);2001(2);	CARC(3);	0-0.5(0);0.5-1.	3	3	3	3	3	3	3	3	3	3	3	3	3	2	2
NARO-HIST202	2002	3	2000(1);2001(2);	CARC(3);	0-0.5(0);0.5-1.	3	3	3	3	3	3	3	3	3	3	3	3	3	0	2
NARO-HIST203	2003	4	2001(1);2002(3);	CARC(3);TARC(1);	0-0.5(0);0.5-1.	4	4	4	4	4	4	4	4	4	4	4	4	4	2	2
NARO-HIST204	2003	4	2001(1);2002(3);	CARC(3);TARC(1);	0-0.5(0);0.5-1.	4	4	4	4	4	4	4	4	4	4	4	4	4	0	1
NARO-HIST205	2003	7	2000(1);2001(2);	CARC(7);	0-0.5(0);0.5-1.	7	7	7	7	7	7	7	7	7	7	7	7	7	3	3
NARO-HIST206	2004	2	2002(1);2003(1);	CARC(2);	0-0.5(0);0.5-1.	2	2	2	2	2	2	2	2	2	2	2	2	2	0	0
NARO-HIST207	2004	4	2002(1);2003(1);	CARC(4);	0-0.5(0);0.5-1.	4	4	4	4	4	4	4	4	4	4	4	4	4	0	0
NARO-HIST208	2004	4	2000(1);2001(1);	CARC(4);	0-0.5(0);0.5-1.	4	4	4	4	4	4	4	4	4	4	4	4	4	2	3
NARO-HIST209	2004	2	2002(1);2003(1);	CARC(2);	0-0.5(0);0.5-1.	2	2	2	2	2	2	2	2	2	2	2	2	2	0	0
NARO-HIST210	2005	3	2002(1);2003(1);	CARC(3);	0-0.5(0);0.5-1.	3	3	3	3	3	3	3	3	3	3	3	3	3	0	0
NARO-HIST211	2005	4	2001(1);2002(1);	CARC(4);	0-0.5(0);0.5-1.	4	4	4	4	4	4	4	4	4	4	4	4	4	1	2
NARO-HIST212	2005	1	2004(1);	CARC(1);	0-0.5(0);0.5-1.	1	1	1	1	1	1	1	1	1	1	1	1	1	1	1
NARO-HIST213	2006	4	2003(1);2004(2);	CARC(3);TARC(1);	0-0.5(0);0.5-1.	4	4	4	4	4	4	4	4	4	4	4	4	4	0	1
NARO-HIST214	2006	3	2004(1);2005(2);	CARC(2);TARC(1);	0-0.5(0);0.5-1.	3	1	1	3	3	3	1	1	1	1	2	2	3	0	0
NARO-HIST215	2006	6	2000(1);2001(1);	CARC(6);	0-0.5(0);0.5-1.	6	6	6	6	6	6	6	6	6	6	6	6	6	0	1
NARO-HIST216	2006	2	2004(1);2005(1);	CARC(2);	0-0.5(0);0.5-1.	2	2	2	2	2	2	2	2	2	2	2	2	2	0	1
NARO-HIST217	2006	1	2005(1);	CARC(1);	0-0.5(0);0.5-1.	1	1	1	1	1	1	1	1	1	1	1	1	1	0	0

NARO-HIST218		2007	3	2004(1);2005(1);	CARC(3);	0-0.5(0);0.5-1.	3	3	3	3	3	3	3	3	3	3	3	3	3	1	1
NARO-HIST219		2007	2	2005(1);2006(1);	CARC(2);	0-0.5(0);0.5-1.	2	2	2	2	2	2	2	2	2	2	2	2	2	1	1
NARO-HIST220		2007	3	2004(1);2005(1);	CARC(3);	0-0.5(0);0.5-1.	3	3	3	3	3	3	3	3	3	3	3	3	3	1	1
NARO-HIST221		2007	3	2005(1);2006(2);	CARC(2);TARC(1);	0-0.5(0);0.5-1.	3	3	3	3	3	3	3	3	3	3	3	3	3	0	1
NARO-HIST222		2007	3	2004(1);2005(1);	CARC(3);	0-0.5(0);0.5-1.	3	3	3	3	3	3	3	3	3	3	3	3	3	1	1
NARO-HIST223		2007	2	2005(1);2006(1);	CARC(2);	0-0.5(0);0.5-1.	2	0	0	2	2	2	0	0	0	0	0	1	2	0	0
NARO-HIST224		2007	2	2005(1);2006(1);	CARC(2);	0-0.5(0);0.5-1.	2	2	2	2	2	2	2	2	2	2	2	2	2	0	0
NARO-HIST225		2007	2	2005(1);2006(1);	CARC(2);	0-0.5(0);0.5-1.	2	2	2	2	2	2	2	2	2	2	2	2	2	1	1
NARO-HIST226		2008	3	2005(1);2006(1);	CARC(3);	0-0.5(0);0.5-1.	3	3	3	3	3	3	3	3	3	3	3	3	3	0	1
NARO-HIST227		2008	3	2005(1);2006(1);	CARC(3);	0-0.5(0);0.5-1.	3	3	3	3	3	3	3	3	3	3	3	3	3	0	1
NARO-HIST228		2008	2	2006(1);2007(1);	CARC(2);	0-0.5(0);0.5-1.	2	2	2	2	2	2	2	2	2	2	2	2	2	0	1
NARO-HIST229		2008	3	2006(1);2007(2);	CARC(2);WARC(1);	0-0.5(0);0.5-1.	3	3	3	3	3	3	3	3	3	3	3	3	3	0	1
NARO-HIST230		2008	6	2007(1);2014(1);	CARC(6);	0-0.5(0);0.5-1.	6	6	6	6	6	6	6	6	6	6	6	6	6	0	1
NARO-HIST231		2008	1	2007(1);	CARC(1);	0-0.5(0);0.5-1.	1	1	1	1	1	1	1	1	1	1	1	1	1	1	1
NARO-HIST232		2009	2	2007(1);2008(1);	CARC(2);	0-0.5(0);0.5-1.	2	2	2	2	2	2	2	2	2	2	2	2	2	0	0
NARO-HIST233		2009	2	2007(1);2008(1);	CARC(2);	0-0.5(0);0.5-1.	2	2	2	2	2	2	2	2	2	2	2	2	2	0	0
NARO-HIST234		2009	3	2006(1);2007(1);	CARC(3);	0-0.5(0);0.5-1.	3	3	3	3	3	3	3	3	3	3	3	3	3	0	0
NARO-HIST235		2009	2	2007(1);2008(1);	CARC(2);	0-0.5(0);0.5-1.	2	2	2	2	2	2	2	2	2	2	2	2	2	0	0
NARO-HIST236		2009	3	2006(1);2007(1);	CARC(3);	0-0.5(0);0.5-1.	3	3	3	3	3	3	3	3	3	3	3	3	3	2	0
NARO-HIST237		2009	4	2006(1);2007(1);	CARC(3);TARC(1);	0-0.5(0);0.5-1.	4	4	4	4	4	4	4	4	4	4	4	4	4	0	0
NARO-HIST238		2009	4	2006(1);2007(2);	CARC(3);TARC(1);	0-0.5(0);0.5-1.	4	1	1	4	4	4	0	0	0	0	0	0	4	0	0
NARO-HIST239		2010	2	2008(1);2009(1);	CARC(2);	0-0.5(0);0.5-1.	2	2	2	2	2	2	2	2	2	2	2	2	2	1	1
NARO-HIST240		2010	3	2007(1);2008(1);	CARC(3);	0-0.5(0);0.5-1.	3	3	3	3	3	3	3	3	3	3	3	3	3	1	1
NARO-HIST241		2010	3	2007(1);2008(1);	CARC(3);	0-0.5(0);0.5-1.	3	3	3	3	3	3	3	3	3	3	3	3	3	0	1
NARO-HIST242		2010	2	2008(1);2009(1);	CARC(2);	0-0.5(0);0.5-1.	2	2	2	2	2	2	2	2	2	2	2	2	2	0	0
NARO-HIST243		2010	3	2008(1);2009(1);	CARC(3);	0-0.5(0);0.5-1.	3	3	3	3	3	3	3	3	3	3	3	3	3	2	2
NARO-HIST244		2010	4	2005(1);2006(1);	CARC(3);TARC(1);	0-0.5(0);0.5-1.	4	2	2	4	4	4	1	1	1	1	1	1	4	0	0
NARO-HIST245		2010	4	2007(1);2008(2);	CARC(3);TARC(1);	0-0.5(0);0.5-1.	4	4	4	4	4	4	4	4	4	4	4	4	4	1	1
NARO-HIST246		2010	5	2007(1);2008(2);	CARC(3);NICS(1);WA	0-0.5(0);0.5-1.	5	3	3	5	5	5	3	3	3	3	3	3	5	2	1
NARO-HIST247		2010	5	2007(2);2008(2);	CARC(3);TARC(2);	0-0.5(0);0.5-1.	5	3	3	5	5	5	3	3	3	3	3	2	5	0	0
NARO-HIST248		2011	1	2010(1);	CARC(1);	0-0.5(0);0.5-1.	1	1	1	1	1	1	1	1	1	1	1	1	1	0	0
NARO-HIST249		2011	2	2009(1);2010(1);	CARC(2);	0-0.5(0);0.5-1.	2	2	2	2	2	2	2	2	2	2	2	2	2	1	1
NARO-HIST250		2011	4	2008(1);2009(1);	CARC(3);NICS(1);	0-0.5(0);0.5-1.	4	4	4	4	4	4	4	4	4	4	4	4	4	1	1
NARO-HIST251		2011	6	2007(1);2008(1);	CARC(6);	0-0.5(0);0.5-1.	6	6	6	6	6	6	6	6	6	6	6	6	6	1	1
NARO-HIST252		2011	3	2009(1);2010(2);	CARC(2);TARC(1);	0-0.5(0);0.5-1.	3	3	3	3	3	3	3	3	3	3	3	3	3	0	1
NARO-HIST253		2012	2	2010(1);2011(1);	CARC(2);	0-0.5(0);0.5-1.	2	2	2	2	2	2	2	2	2	2	2	2	2	1	1
NARO-HIST254		2012	3	2009(1);2010(1);	CARC(3);	0-0.5(0);0.5-1.	3	3	3	3	3	3	3	3	3	3	3	3	3	2	1
NARO-HIST255		2012	2	2010(1);2011(1);	CARC(2);	0-0.5(0);0.5-1.	2	2	2	2	2	2	2	2	2	2	2	2	2	1	1
NARO-HIST256		2012	3	2010(1);2011(2);	CARC(2);WARC(1);	0-0.5(0);0.5-1.	3	3	3	3	3	3	2	3	2	2	3	3	3	1	1
NARO-HIST257		2012	1	2011(1);	CARC(1);	0-0.5(0);0.5-1.	1	1	1	1	1	1	1	1	1	1	1	1	1	1	0
NARO-HIST258		2012	3	2009(1);2010(2);	CARC(2);TARC(1);	0-0.5(0);0.5-1.	3	3	3	3	3	3	3	3	3	3	3	3	3	1	1
NARO-HIST259		2013	3	2011(1);2012(2);	CARC(2);TARC(1);	0-0.5(0);0.5-1.	3	3	3	3	3	3	3	3	3	3	3	3	3	1	1
NARO-HIST260		2013	2	2011(1);2012(1);	CARC(2);	0-0.5(0);0.5-1.	2	2	2	2	2	2	2	2	2	2	2	2	2	1	1
NARO-HIST261		2013	2	2011(1);2012(1);	CARC(2);	0-0.5(0);0.5-1.	2	2	2	2	2	2	2	2	2	2	2	2	2	1	1
NARO-HIST262		2013	2	2011(1);2012(1);	CARC(2);	0-0.5(0);0.5-1.	2	2	2	2	2	2	2	2	2	2	2	2	2	1	1
NARO-HIST263		2014	2	2012(1);2013(1);	CARC(2);	0-0.5(0);0.5-1.	2	2	2	2	2	2	2	2	2	2	2	2	2	1	1
NARO-HIST264		2014	6	2009(1);2010(1);	CARC(6);	0-0.5(0);0.5-1.	6	6	6	6	6	6	6	6	6	6	6	6	6	1	1
NARO-HIST265		2014	4	2011(1);2012(1);	CARC(4);	0-0.5(0);0.5-1.	4	4	4	4	4	4	4	4	4	4	4	4	4	3	1
NARO-HIST266		2014	4	2011(1);2012(1);	CARC(4);	0-0.5(0);0.5-1.	4	4	4	4	4	4	4	4	4	4	4	4	4	1	1
NARO-HIST267		2015	3	2013(1);2014(2);	CARC(3);	0-0.5(0);0.5-1.	3	3	3	3	3	3	3	3	3	3	3	3	3	0	0
NARO-HIST268		2015	3	2013(1);2014(2);	CARC(3);	0-0.5(0);0.5-1.	3	3	3	3	3	3	3	3	3	3	3	3	3	0	0
NARO-HIST269		2015	4	2013(1);2014(3);	CARC(3);NICS(1);	0-0.5(0);0.5-1.	4	4	4	4	4	4	4	4	4	4	4	4	4	0	0
NARO-HIST270		2015	4	2012(1);2013(1);	CARC(4);	0-0.5(0);0.5-1.	4	4	4	4	4	4	4	4	4	4	4	4	4	3	0
NARO-HIST271		2015	6	2011(1);2012(1);	CARC(6);	0-0.5(0);0.5-1.	6	6	6	6	6	6	6	6	6	6	6	6	6	1	0
NARO-HIST272		2016	3	2014(1);2015(2);	CARC(3);	0-0.5(0);0.5-1.	3	3	3	3	3	3	3	3	3	3	3	3	3	1	1
NARO-HIST273		2016	4	2014(1);2015(3);	CARC(3);NICS(1);	0-0.5(0);0.5-1.	4	4	4	4	4	4	4	4	4	4	4	4	4	1	1
NARO-HIST274		2016	2	2014(1);2015(1);	CARC(2);	0-0.5(0);0.5-1.	2	2	2	2	2	2	2	2	2	2	2	2	2	0	0
NARO-HIST275		2017	3	2015(1);2016(2);	CARC(3);	0-0.5(0);0.5-1.	3	3	3	3	3	3	3	3	3	3	3	3	3	1	0
NARO-HIST276		2017	6	2014(1);2015(2);	CARC(5);TARC(1);	0-0.5(0);0.5-1.	6	6	6	6	6	6	6	6	6	6	6	6	6	1	0
NARO-HIST277		2017	4	2015(1);2016(3);	CARC(3);NICS(1);	0-0.5(0);0.5-1.	4	4	4	4	4	4	4	4	4	4	4	4	4	1	0
NARO-HIST278		2017	3	2015(1);2016(2);	CARC(3);	0-0.5(0);0.5-1.	3	3	3	3	3	3	3	3	3	3	3	3	3	1	0
NARO-HIST279		2017	3	2015(1);2016(2);	CARC(3);	0-0.5(0);0.5-1.	3	3	3	3	3	3	3	3	3	3	3	3	3	1	0
NARO-HIST280		2017	11	2013(1);2014(2);	CARC(9);NICS(2);	0-0.5(0);0.5-1.	11	11	11	11	11	11	11	11	11	11	11	11	11	1	0
NARO-HIST281		2017	6	2014(1);2015(2);	CARC(3);NICS(1);TAR	0-0.5(0);0.5-1.	6	1	1	6	6	6	2	3	2	2	4	2	6	0	0
NARO-HIST282		2017	1	2016(1);	CARC(1);	0-0.5(0);0.5-1.	1	0	0	1	1	1	1	1	1	1	1	1	1	0	0
NARO-HIST283		2018	3	2016(1);2017(2);	CARC(3);	0-0.5(0);0.5-1.	3	3	3	3	3	3	3	3	3	3	3	3	3	0	0
NARO-HIST284	NA		3	2017(1);2018(2);	CARC(3);	0-0.5(0);0.5-1.	3	3	3	3	3	3	3	3	3	3	3	3	3	1	0
NARO-HIST285	NA		2	2017(1);2018(1);	CARC(2);	0-0.5(0);0.5-1.	2	2	2	2	2	2	2	2	2	2	2	2	2	1	0
NARO-HIST286	NA		2	2017(1);2018(1);	CARC(2);	0-0.5(0);0.5-1.	2	2	2	2	2	2	2	2	2	2	2	2	2	0	0
NARO-HIST287	NA		3	2017(1);2018(2);	CARC(3);	0-0.5(0);0.5-1.	3	3	3	3	3	3	3	3	3	3	3	3	3	1	1
NARO-HIST288		2000	3	1998(1);1999(2);	CARC(3);	0-0.5(0);0.5-1.	3	3	3	3	3	3	3	3	3	3	3	3	3	2	2
NARO-HIST289		2000	11	1998(1);1999(2);	NICS(5);WARC(6);	0-0.5(0);0.5-1.	11	11	11	11	11	11	10	10	10	10	10	10	11	0	0
NARO-HIST290		2001	1	2004(1);	NICS(1);	0-0.5(0);0.5-1.	1	1	1	1	1	1	1	1	1	1	1	1	1	0	0
NARO-HIST291		2001	9	2004(3);2005(3);	NICS(3);WARC(6);	0-0.5(1);0.5-1.	9	9	9	9	9	9	9	9	9	9	9	9	9	2	2
NARO-HIST292	NA		1	1996(1);	WARC(1);	0-0.5(0);0.5-1.	1	1	1	1	1	1	1	1	1	1	1	1	1	0	0
NARO-HIST293		1982	4	2000(1);2001(1);	WARC(4);	0-0.5(0);0.5-1.	4	4	4	4	4	4	4	4	4	4	4	4	4	0	0
NARO-HIST294	NA		2	2002(1);2003(1);	KARC(2);	0-0.5(0);0.5-1.	2	2	2	2	2	2	2	2	2	2	2	2	2	0	0
NARO-HIST295		1985	86	1996(1);1997(1);	HARC(86);	0-0.5(3);0.5-1.	86	81	81	86	86	86	60	82	60	60	78	73	86	44	53
NARO-HIST296		1990	8	1997(1);1998(2);	HARC(8);	0-0.5(0);0.5-1.	8	8	8	8	8	8	8	8	8	8	8	8	8	3	3
NARO-HIST297		1992	6	1996(1);2000(1);	HARC(6);	0-0.5(0);0.5-1.	6	6	6	6	6	6	3	6	3	3	6	6	6	0	1
NARO-HIST298		1993	33	1997(1);1998(2);	HARC(33);	0-0.5(0);0.5-1.	33	33	33	33	33	33	32	33	32	32	33	33	32	24	28
NARO-HIST299		1998	3	2012(2);2013(1);	HARC(3);	0-0.5(0);0.5-1.	3	3	3	3	3	3	3	3	3	3	3	0	3	3	3
NARO-HIST300		1999	21	2006(1);2007(4);	HARC(21);	0-0.5(0);0.5-1.	21	20	20	21	21	21	13	21	13	13	20	21	21	13	14
NARO-HIST301		2002	16	2006(3);2007(2);	HARC(16);	0-0.5(0);0.5-1.	16	16	16	16	16	16	16	16	16	16	16	16	16	15	16
NARO-HIST302		1960	6	1995(1);1996(2);	CARC(4);NICS(2);	0-0.5(0);0.5-1.	5	5	5	5	5	5	5	5	5	5	5	5	5	0	1
NARO-HIST303		1961	5	1993(1);1994(2);	CARC(5);	0-0.5(0);0.5-1.	5	5	5	5	5	5	5	5	5	5	5	5	5	0	0
NARO-HIST304		1969	2	1998(1);1999(1);	WARC(2);	0-0.5(0);0.5-1.	2	2	2	2	2	2	2	2	2	2	2	2	2	0	0
NARO-HIST305		1973	7	1996(3);1998(1);	CARC(7);	0-0.5(0);0.5-1.	5	5	5	5	5	5	5	5	5	5	5	5	5	3	3
NARO-HIST306		1987	27	1998(1);1999(2);	NICS(18);WARC(9);	0-0.5(0);0.5-1.	27	27	27	27	27	27	22	23	22	18	24	24	26	1	1
NARO-HIST307		1987	22	1994(3);1995(3);	CARC(3);KARC(7);NIC	0-0.5(0);0.5-1.	22	22	22	22	22	22	22	22	22	22	22	21	22	0	1
NARO-HIST308		1991	9	1995(1);1996(2);	CARC(3);NICS(6);	0-0.5(0);0.5-1.	9	9	9	9	9	9	9	9	9	9	9	9	9	0	0
NARO-HIST309		1992	17	1995(1);2003(1);	CARC(5);KARC(1);NIC	0-0.5(1);0.5-1.	17	17	17	17	17	17	17	16	17	16	17	17	17	12	4
NARO-HIST310		1995	2	2011(1);2012(1);	WARC(2);	0-0.5(0);0.5-1.	2	1	1	2	2	2	0	2	0	0	2	2	2	1	0
NARO-HIST311		1996	2	1994(1);1995(1);	NICS(2);	0-0.5(0);0.5-1.	2	1	1	2	2	2	2	2	2	2	2	2	2	0	0
NARO-HIST312		1996	2	1994(1);1995(1);	NICS(2);	0-0.5(0);0.5-1.	2	1	1	2	2	2	2	2	2	2	2	2	2	0	0
NARO-HIST313		1996	2	1994(1);1995(1);	NICS(2);	0-0.5(0);0.5-1.	2	1	1	2	2	2	2	2	2	2	2	2	2	0	0
NARO-HIST314		1996	3	1994(1);1995(2);	NICS(2);WARC(1);	0-0.5(0);0.5-1.	3	1	1	3	3	3	3	2	3	2	3	3	3	0	0
NARO-HIST315		1996	1	1995(1);	NICS(1);	0-0.5(0);0.5-1.	1	1	1	1	1	1	1	1	1	1	1	1	1	1	0
NARO-HIST316		1996	8	1994(1);1995(2);	NICS(8);	0-0.5(0);0.5-1.	8	8	8	8	8	8	8	5	8	5	8	8	8	0	0
NARO-HIST317		1996	2	1994(1);1995(1);	NICS(2);	0-0.5(0);0.5-1.	2	2	2	2	2	2	2	2	2	2	2	2	2	0	0
NARO-HIST318		1996	2	1994(1);1995(1);	NICS(2);	0-0.5(0);0.5-1.	2	2	2	2	2	2	2	2	2	2	2	2	2	0	0
NARO-HIST319		1997	4	1995(2);1996(2);	NICS(4);	0-0.5(0);0.5-1.	4	4	4	4	4	4	4	0	4	0	4	4	4	0	0
NARO-HIST320		1997	3	1995(1);1996(2);	NICS(2);WARC(1);	0-0.5(0);0.5-1.	3	2	2	3	3	3	3	0	3	0	3	3	3	0	0
NARO-HIST321		1997	1	1996(1);	NICS(1);	0-0.5(0);0.5-1.	1	1	1	1	1	1	1	1	1	1	1	1	1	0	0
NARO-HIST322		1997	5	1996(1);2003(1);	CARC(1);NICS(4);	0-0.5(0);0.5-1.	5	5	5	5	5	5	5	5	5	5	5	5	5	2	2
NARO-HIST323		1997	1	1996(1);	NICS(1);	0-0.5(0);0.5-1.	1	1	1	1	1	1	0	1	1	1	0	1	1	0	0
NARO-HIST324		1998	3	1996(1);1997(2);	NICS(3);	0-0.5(0);0.5-1.	3	3	3	3	3	3	3	3	3	3	3	3	3	0	0
NARO-HIST325		1998	5	1995(1);1996(1);	NICS(4);WARC(1);	0-0.5(0);0.5-1.	5	5	5	5	5	5	5	5	5	5	5	5	5	0	0
NARO-HIST326		1998	2	1996(1);1997(1);	NICS(2);	0-0.5(0);0.5-1.	2	2	2	2	2	2	2	2	2	2	2	2	1	0	0
NARO-HIST327		1999	3	1997(1);1998(2);	NICS(3);	0-0.5(0);0.5-1.	3	3	3	3	3	3	3	3	3	3	3	3	3	0	0
NARO-HIST328		1999	3	1997(1);1998(2);	NICS(3);	0-0.5(0);0.5-1.	3	3	3	3	3	3	3	3	3	3	3	3	3	0	0

NARO-HIST329		1999	3	1997(1);1998(2);	NICS(3);	0-0.5(0);0.5-1.	3	3	3	3	3	3	3	3	3	3	3	3	3	0	0
NARO-HIST330		1999	5	1996(1);1997(2);	NICS(5);	0-0.5(0);0.5-1.	5	5	5	5	5	5	5	5	5	5	5	5	5	0	0
NARO-HIST331		2000	3	1998(1);1999(2);	NICS(3);	0-0.5(0);0.5-1.	3	3	3	3	3	3	3	3	3	3	3	3	3	0	0
NARO-HIST332		2000	5	1997(1);1998(2);	NICS(5);	0-0.5(0);0.5-1.	5	5	5	5	5	5	5	5	5	5	5	5	5	0	0
NARO-HIST333		2000	5	1997(1);1998(2);	NICS(5);	0-0.5(0);0.5-1.	5	5	5	5	5	5	5	5	5	5	5	5	5	0	0
NARO-HIST334		2000	24	1998(1);1999(2);	NICS(21);WARC(3);	0-0.5(0);0.5-1.	24	24	24	24	24	24	20	24	20	20	20	19	24	0	0
NARO-HIST335		2000	1	1997(1);	NICS(1);	0-0.5(0);0.5-1.	1	1	1	1	1	1	1	1	1	1	1	1	1	0	0
NARO-HIST336		2001	5	1998(1);1999(2);	NICS(5);	0-0.5(0);0.5-1.	5	5	5	5	5	5	5	5	5	5	5	5	5	0	0
NARO-HIST337		2001	12	1999(1);2000(2);	NICS(12);	0-0.5(0);0.5-1.	12	12	12	12	12	12	12	12	12	12	12	12	12	0	0
NARO-HIST338		2001	5	1998(1);1999(2);	NICS(5);	0-0.5(0);0.5-1.	5	5	5	5	5	5	5	5	5	5	5	5	5	0	0
NARO-HIST339		2001	3	1999(1);2000(2);	NICS(3);	0-0.5(0);0.5-1.	3	3	3	3	3	3	3	3	3	3	3	3	3	0	0
NARO-HIST340		2002	7	1998(1);1999(2);	NICS(7);	0-0.5(0);0.5-1.	7	7	7	7	7	7	7	7	7	7	7	7	7	0	0
NARO-HIST341		2002	5	1999(1);2000(2);	NICS(5);	0-0.5(0);0.5-1.	5	5	5	5	5	5	5	5	5	5	5	5	5	0	0
NARO-HIST342		2002	4	2000(1);2001(3);	NICS(3);WARC(1);	0-0.5(0);0.5-1.	4	4	4	4	4	4	4	4	4	4	4	4	4	0	0
NARO-HIST343		2002	9	1998(1);1999(1);	NICS(8);WARC(1);	0-0.5(0);0.5-1.	9	9	9	9	9	9	5	6	5	3	6	5	9	0	0
NARO-HIST344		2002	5	1999(1);2000(2);	NICS(5);	0-0.5(0);0.5-1.	5	5	5	5	5	5	4	4	4	4	4	4	5	0	0
NARO-HIST345		2003	3	2001(1);2002(2);	NICS(3);	0-0.5(0);0.5-1.	3	3	3	3	3	3	3	3	3	3	3	3	3	0	0
NARO-HIST346		2003	3	2001(1);2002(2);	NICS(3);	0-0.5(0);0.5-1.	3	3	3	3	3	3	3	3	3	3	3	3	3	0	0
NARO-HIST347		2003	1	2002(1);	NICS(1);	0-0.5(0);0.5-1.	1	1	1	1	1	1	1	1	1	1	1	1	1	0	0
NARO-HIST348		2004	5	2001(1);2002(2);	NICS(5);	0-0.5(0);0.5-1.	5	5	5	5	5	5	5	5	5	5	5	5	5	0	0
NARO-HIST349		2005	45	2003(1);2004(2);	CARC(17);KARC(2);N	0-0.5(0);0.5-1.	44	44	44	44	44	44	43	44	43	43	44	44	44	3	3
NARO-HIST350		2005	3	2003(1);2004(2);	NICS(3);	0-0.5(0);0.5-1.	3	3	3	3	3	3	3	3	3	3	3	3	3	0	0
NARO-HIST351		2005	4	2003(1);2004(2);	KARC(1);NICS(3);	0-0.5(0);0.5-1.	4	4	4	4	4	4	4	4	4	4	4	4	4	2	2
NARO-HIST352		2005	10	2004(1);2009(2);	NICS(8);WARC(2);	0-0.5(0);0.5-1.	10	8	8	10	10	10	8	2	8	2	9	9	10	0	0
NARO-HIST353		2005	10	2003(1);2004(1);	CARC(6);KARC(1);NIC	0-0.5(0);0.5-1.	10	10	10	10	10	10	9	10	9	9	10	10	10	1	0
NARO-HIST354		2006	3	2004(2);2005(1);	NICS(3);	0-0.5(0);0.5-1.	3	3	3	3	3	3	3	3	3	3	3	3	3	0	0
NARO-HIST355		2006	3	2004(1);2005(2);	NICS(3);	0-0.5(0);0.5-1.	3	3	3	3	3	3	3	3	3	3	3	3	3	0	0
NARO-HIST356		2006	6	2003(1);2004(3);	CARC(1);NICS(4);WA	0-0.5(0);0.5-1.	6	6	6	6	6	6	6	6	6	6	6	6	6	0	0
NARO-HIST357		2006	5	2004(1);2005(2);	NICS(3);WARC(2);	0-0.5(1);0.5-1.	5	5	5	5	5	5	5	5	5	5	5	5	5	2	2
NARO-HIST358		2006	3	2003(1);2004(1);	NICS(3);	0-0.5(0);0.5-1.	3	3	3	3	3	3	3	3	3	3	3	3	3	0	0
NARO-HIST359		2007	3	2005(1);2006(2);	NICS(3);	0-0.5(0);0.5-1.	3	3	3	3	3	3	3	3	3	3	3	3	3	0	0
NARO-HIST360		2007	3	2005(1);2006(2);	NICS(3);	0-0.5(0);0.5-1.	3	3	3	3	3	3	3	3	3	3	3	3	3	0	0
NARO-HIST361		2007	4	2005(1);2006(3);	NICS(3);WARC(1);	0-0.5(0);0.5-1.	4	3	3	4	4	4	4	4	4	4	4	4	4	0	0
NARO-HIST362		2007	5	2003(1);2004(1);	NICS(5);	0-0.5(0);0.5-1.	5	5	5	5	5	5	5	5	5	5	5	5	5	0	0
NARO-HIST363		2007	7	2001(1);2002(1);	NICS(7);	0-0.5(0);0.5-1.	7	7	7	7	7	7	7	7	7	7	7	7	7	0	0
NARO-HIST364		2007	1	2006(1);	NICS(1);	0-0.5(0);0.5-1.	1	1	1	1	1	1	1	1	1	1	1	1	1	0	0
NARO-HIST365		2008	15	2006(1);2007(2);	CARC(1);NICS(14);	0-0.5(0);0.5-1.	15	15	15	15	15	15	15	15	15	15	15	15	15	1	0
NARO-HIST366		2008	22	2006(1);2007(4);	KARC(3);NICS(16);W	0-0.5(0);0.5-1.	22	22	22	22	22	22	22	21	22	21	22	22	22	1	1
NARO-HIST367		2008	5	2005(1);2006(2);	NICS(5);	0-0.5(0);0.5-1.	5	5	5	5	5	5	5	5	5	5	5	5	5	0	0
NARO-HIST368		2008	2	2006(1);2007(1);	NICS(2);	0-0.5(0);0.5-1.	2	2	2	2	2	2	2	2	2	2	2	2	2	0	0
NARO-HIST369		2008	11	2006(1);2007(2);	NICS(11);	0-0.5(0);0.5-1.	11	11	11	11	11	11	11	11	11	11	11	11	11	0	0
NARO-HIST370		2008	12	2007(2);2008(2);	NICS(12);	0-0.5(0);0.5-1.	12	12	12	12	12	12	12	11	12	11	12	12	12	0	0
NARO-HIST371		2009	13	2006(1);2007(2);	NICS(13);	0-0.5(0);0.5-1.	13	13	13	13	13	13	13	13	13	13	13	13	13	1	0
NARO-HIST372		2009	6	2005(1);2006(2);	NICS(6);	0-0.5(0);0.5-1.	6	6	6	6	6	6	6	6	6	6	6	6	6	4	0
NARO-HIST373		2009	3	2007(1);2008(2);	NICS(3);	0-0.5(0);0.5-1.	3	3	3	3	3	3	3	3	3	3	3	3	3	0	0
NARO-HIST374		2009	3	2007(1);2008(2);	NICS(3);	0-0.5(0);0.5-1.	3	3	3	3	3	3	3	3	3	3	3	3	3	0	0
NARO-HIST375		2009	8	2005(1);2006(2);	NICS(7);WARC(1);	0-0.5(0);0.5-1.	8	8	8	8	8	8	8	8	8	8	8	8	8	0	0
NARO-HIST376		2009	3	2007(1);2008(2);	NICS(3);	0-0.5(0);0.5-1.	3	3	3	3	3	3	3	3	3	3	3	3	3	0	0
NARO-HIST377		2010	3	2008(1);2009(2);	NICS(3);	0-0.5(0);0.5-1.	3	3	3	3	3	3	3	3	3	3	3	3	3	0	0
NARO-HIST378		2010	4	2007(1);2008(1);	NICS(4);	0-0.5(0);0.5-1.	4	4	4	4	4	4	4	4	4	4	4	4	4	0	0
NARO-HIST379		2010	4	2008(1);2009(3);	NICS(3);WARC(1);	0-0.5(0);0.5-1.	4	4	4	4	4	4	4	3	4	3	4	4	4	0	0
NARO-HIST380		2010	16	2005(1);2006(2);	CARC(1);NICS(11);TA	0-0.5(0);0.5-1.	16	14	14	16	16	16	12	12	12	12	12	13	16	0	0
NARO-HIST381		2010	2	2008(1);2009(1);	NICS(1);TARC(1);	0-0.5(0);0.5-1.	2	1	1	2	2	2	1	0	1	0	1	1	2	0	0
NARO-HIST382		2011	4	2008(1);2009(2);	NICS(4);	0-0.5(0);0.5-1.	4	4	4	4	4	4	4	4	4	4	4	4	4	0	0
NARO-HIST383		2011	5	2008(1);2009(2);	NICS(5);	0-0.5(0);0.5-1.	5	5	5	5	5	5	5	5	5	5	5	5	5	0	0
NARO-HIST384		2011	3	2009(1);2010(2);	NICS(3);	0-0.5(0);0.5-1.	3	3	3	3	3	3	3	0	3	0	3	3	3	0	0
NARO-HIST385		2012	4	2010(1);2011(3);	NICS(3);WARC(1);	0-0.5(0);0.5-1.	4	3	3	4	4	4	4	4	4	4	4	4	4	0	0
NARO-HIST386		2013	9	2011(1);2012(2);	NICS(7);WARC(2);	0-0.5(0);0.5-1.	9	9	9	9	9	9	9	9	9	9	9	9	9	1	1
NARO-HIST387		2013	3	2011(1);2012(2);	NICS(3);	0-0.5(0);0.5-1.	3	3	3	3	3	3	3	3	3	3	3	3	3	0	0
NARO-HIST388		2013	6	2010(1);2011(3);	NICS(5);WARC(1);	0-0.5(0);0.5-1.	6	6	6	6	6	6	6	6	6	6	6	6	6	0	0
NARO-HIST389		2013	3	2011(1);2012(2);	NICS(3);	0-0.5(0);0.5-1.	3	3	3	3	3	3	3	3	3	3	3	3	3	0	0
NARO-HIST390		2013	7	2011(1);2012(2);	NICS(7);	0-0.5(0);0.5-1.	7	7	7	7	7	7	7	0	7	0	7	7	7	0	0
NARO-HIST391		2013	1	2012(1);	NICS(1);	0-0.5(0);0.5-1.	1	1	1	1	1	1	1	0	1	0	1	1	1	0	0
NARO-HIST392		2014	4	2012(1);2013(3);	NICS(3);WARC(1);	0-0.5(0);0.5-1.	4	4	4	4	4	4	4	4	4	4	4	4	4	0	0
NARO-HIST393		2014	4	2012(1);2013(3);	NICS(3);WARC(1);	0-0.5(0);0.5-1.	4	4	4	4	4	4	4	4	4	4	4	4	4	0	0
NARO-HIST394		2014	3	2012(1);2013(2);	NICS(3);	0-0.5(0);0.5-1.	3	3	3	3	3	3	3	3	3	3	3	3	3	0	0
NARO-HIST395		2014	3	2012(1);2013(2);	NICS(3);	0-0.5(0);0.5-1.	3	3	3	3	3	3	3	3	3	3	3	3	3	0	0
NARO-HIST396		2014	3	2012(1);2013(2);	NICS(3);	0-0.5(0);0.5-1.	3	3	3	3	3	3	3	3	3	3	3	3	3	0	0
NARO-HIST397		2014	12	2007(2);2008(2);	NICS(12);	0-0.5(0);0.5-1.	12	12	12	12	12	12	12	12	12	12	12	12	12	0	0
NARO-HIST398		2014	11	2006(2);2007(3);	NICS(11);	0-0.5(0);0.5-1.	11	11	11	11	11	11	11	11	11	11	11	11	11	0	0
NARO-HIST399		2015	4	2013(1);2014(3);	CARC(1);NICS(3);	0-0.5(0);0.5-1.	4	4	4	4	4	4	4	4	4	4	4	4	4	0	0
NARO-HIST400		2015	3	2013(1);2014(2);	NICS(3);	0-0.5(0);0.5-1.	3	3	3	3	3	3	3	3	3	3	3	3	3	0	0
NARO-HIST401		2015	6	2013(1);2014(3);	NICS(5);WARC(1);	0-0.5(0);0.5-1.	6	6	6	6	6	6	6	6	6	6	6	6	6	0	0
NARO-HIST402		2015	4	2013(1);2014(3);	KARC(1);NICS(3);	0-0.5(0);0.5-1.	4	4	4	4	4	4	4	4	4	4	4	4	4	0	0
NARO-HIST403		2015	4	2013(1);2014(3);	KARC(1);NICS(3);	0-0.5(0);0.5-1.	4	4	4	4	4	4	4	4	4	4	4	4	4	0	0
NARO-HIST404		2016	4	2014(2);2015(2);	NICS(4);	0-0.5(0);0.5-1.	4	4	4	4	4	4	4	4	4	4	4	4	4	0	0
NARO-HIST405		2016	5	2013(1);2014(2);	NICS(5);	0-0.5(0);0.5-1.	5	5	5	5	5	5	5	5	5	5	5	5	5	0	0
NARO-HIST406		2016	3	2014(1);2015(2);	NICS(3);	0-0.5(0);0.5-1.	3	3	3	3	3	3	3	3	3	3	3	3	3	0	0
NARO-HIST407		2016	6	2013(1);2014(3);	NICS(5);WARC(1);	0-0.5(0);0.5-1.	6	6	6	6	6	6	6	6	6	6	6	6	6	0	0
NARO-HIST408		2016	8	2012(1);2013(2);	NICS(8);	0-0.5(0);0.5-1.	8	8	8	8	8	8	8	8	8	8	8	8	8	0	0
NARO-HIST409		2017	3	2015(1);2016(2);	NICS(3);	0-0.5(0);0.5-1.	3	3	3	3	3	3	3	3	3	3	3	3	3	0	0
NARO-HIST410		2017	2	2015(1);2016(1);	NICS(2);	0-0.5(0);0.5-1.	2	2	2	2	2	2	2	0	2	0	2	2	2	0	0
NARO-HIST411		2018	5	2015(1);2016(2);	NICS(5);	0-0.5(0);0.5-1.	5	5	5	5	5	5	5	5	5	5	5	5	5	1	0
NARO-HIST412		2018	3	2016(1);2017(2);	NICS(3);	0-0.5(0);0.5-1.	3	3	3	3	3	3	3	3	3	3	3	3	3	1	1
NARO-HIST413		2018	3	2016(1);2017(2);	NICS(3);	0-0.5(0);0.5-1.	3	3	3	3	3	3	3	3	3	3	3	3	3	0	0
NARO-HIST414		2019	7	2016(3);2017(2);	KARC(1);NICS(6);	0-0.5(0);0.5-1.	7	7	7	7	7	7	7	7	7	7	7	7	7	0	0
NARO-HIST415		2019	6	2016(1);2017(3);	KARC(1);NICS(5);	0-0.5(0);0.5-1.	6	6	6	6	6	6	6	6	6	6	6	6	6	0	0
NARO-HIST416		2005	4	2003(1);2004(3);	NICS(3);WARC(1);	0-0.5(0);0.5-1.	4	4	4	4	4	4	4	4	4	4	4	4	4	0	0
NARO-HIST417		2011	3	2009(1);2010(2);	NICS(3);	0-0.5(0);0.5-1.	3	3	3	3	3	3	3	3	3	3	3	3	3	0	0
NARO-HIST418		1986	5	1996(1);1997(1);	HARC(5);	0-0.5(0);0.5-1.	5	5	5	5	5	5	5	5	5	5	5	5	5	0	2
NARO-HIST419		1969	9	2004(1);2005(1);	KARC(9);	0-0.5(0);0.5-1.	9	9	9	9	8	9	9	9	9	9	9	8	9	0	2
NARO-HIST420		1973	52	1993(2);1994(3);	NICS(45);WARC(7);	0-0.5(0);0.5-1.	51	51	50	52	52	52	52	50	52	50	52	52	52	0	0
NARO-HIST421		1974	9	1994(1);1995(1);	KARC(9);	0-0.5(0);0.5-1.	8	8	8	8	8	8	8	9	9	9	9	9	8	1	1
NARO-HIST422		1975	18	1998(1);2001(3);	KARC(18);	0-0.5(0);0.5-1.	18	15	15	18	18	18	7	17	7	7	13	13	18	0	0
NARO-HIST423		1978	11	2001(2);2002(3);	KARC(11);	0-0.5(0);0.5-1.	11	5	5	11	11	11	0	11	0	0	5	5	11	0	0
NARO-HIST424		1986	166	1992(1);1993(3);	KARC(43);NICS(50);W	0-0.5(0);0.5-1.	165	163	163	164	164	164	162	161	162	159	164	164	165	19	10
NARO-HIST425	NA		8	2000(1);2001(1);	CARC(8);	0-0.5(0);0.5-1.	8	8	8	8	8	8	8	8	8	8	8	8	8	4	5
NARO-HIST426	NA		18	1994(1);1995(1);	CARC(10);NICS(5);TA	0-0.5(0);0.5-1.	18	18	18	18	18	18	18	16	18	16	18	18	18	0	3
NARO-HIST427		1984	1	1997(1);	CARC(1);	0-0.5(0);0.5-1.	1	1	1	1	1	1	1	1	1	1	1	1	1	1	1
NARO-HIST428		1988	1	2010(1);	CARC(1);	0-0.5(0);0.5-1.	1	1	1	1	1	1	1	1	1	1	1	1	1	0	1
NARO-HIST429		1990	5	1996(2);1997(1);	CARC(5);	0-0.5(0);0.5-1.	5	5	5	5	5	5	5	5	5	5	5	5	5	2	3
NARO-HIST430		1997	1	2015(1);	CARC(1);	0-0.5(0);0.5-1.	1	1	1	1	1	1	1	1	1	1	1	1	1	0	0
NARO-HIST431		2001	2	2012(1);2015(1);	CARC(2);	0-0.5(0);0.5-1.	2	2	2	2	2	2	2	2	2	2	2	2	2	0	0
NARO-HIST432		2012	3	2017(1);2018(2);	CARC(3);	0-0.5(0);0.5-1.	3	3	3	3	3	3	3	3	3	3	3	3	3	0	0
NARO-HIST433		1966	10	1992(1);1994(2);	TARC(10);	0-0.5(0);0.5-1.	10	9	9	10	10	10	10	0	10	0	10	10	9	0	0
NARO-HIST434		1966	12	1994(2);1995(4);	CARC(2);TARC(10);	0-0.5(0);0.5-1.	12	12	12	12	12	12	12	0	12	0	12	12	12	0	0
NARO-HIST435		1969	29	1995(1);1996(1);	CARC(9);TARC(19);W	0-0.5(0);0.5-1.	28	28	28	28	28	28	28	19	28	19	28	27	28	0	4
NARO-HIST436		1988	67	1996(2);1997(2);	CARC(16);TARC(51);	0-0.5(0);0.5-1.	66	59	59	66	66	66	42	26	42	23	60	57	66	7	11
NARO-HIST437		1992	8	1999(1);2005(1);	CARC(2);TARC(6);	0-0.5(0);0.5-1.	8	8	8	8	8	8	8	4	8	4	8	8	8	3	2
NARO-HIST438		1993	14	1995(1);1996(1);	NICS(1);TARC(13);	0-0.5(0);0.5-1.	14	14	14	14	14	14	14	10	14	10	14	14	14	0	0
NARO-HIST439		1995	5	2007(1);2008(1);	CARC(3);TARC(2);	0-0.5(0);0.5-1.	5	5	5	5	5	5	5	3	5	3	5	5	5	1	1

NARO-HIST440		1996	2	1994(1);1995(1);	TARC(2);	0-0.5(0);0.5-1.	2	2	2	2	2	2	2	0	2	0	2	2	2	0	0
NARO-HIST441		1996	2	1994(1);1995(1);	TARC(2);	0-0.5(0);0.5-1.	2	1	1	2	2	2	2	0	2	0	2	2	1	0	0
NARO-HIST442		1996	2	1994(1);1995(1);	TARC(2);	0-0.5(0);0.5-1.	2	1	1	2	2	2	2	0	2	0	2	2	1	0	0
NARO-HIST443		1996	3	1994(1);1995(2);	CARC(1);TARC(2);	0-0.5(0);0.5-1.	3	2	2	3	3	3	3	0	3	0	3	3	2	0	0
NARO-HIST444		1996	1	1992(1);	TARC(1);	0-0.5(0);0.5-1.	1	0	0	1	1	1	1	0	1	0	1	1	1	0	0
NARO-HIST445		1997	2	1994(1);1995(1);	TARC(2);	0-0.5(0);0.5-1.	2	1	1	2	2	2	2	0	2	0	2	2	2	0	0
NARO-HIST446		1997	4	1994(1);1995(2);	CARC(1);TARC(3);	0-0.5(0);0.5-1.	4	4	4	4	4	4	4	0	4	0	4	4	4	0	0
NARO-HIST447		1997	3	1995(1);1996(2);	CARC(1);TARC(2);	0-0.5(0);0.5-1.	3	3	3	3	3	3	3	0	3	0	3	3	3	0	0
NARO-HIST448		1997	2	1995(1);1996(1);	TARC(2);	0-0.5(0);0.5-1.	2	2	2	2	2	2	2	0	2	0	2	2	2	0	0
NARO-HIST449		1998	2	1997(1);1998(1);	TARC(2);	0-0.5(0);0.5-1.	2	2	2	2	2	2	2	0	2	0	2	2	2	0	0
NARO-HIST450		1999	2	1996(1);2002(1);	TARC(2);	0-0.5(0);0.5-1.	2	2	2	2	2	2	2	1	2	1	2	2	2	0	0
NARO-HIST451		1999	2	1997(1);1998(1);	TARC(2);	0-0.5(0);0.5-1.	2	2	2	2	2	2	2	0	2	0	2	2	2	0	0
NARO-HIST452		2000	3	1997(1);1998(1);	TARC(3);	0-0.5(0);0.5-1.	3	3	3	3	3	3	3	0	3	0	3	3	3	0	0
NARO-HIST453		2000	2	1998(1);1999(1);	TARC(2);	0-0.5(0);0.5-1.	2	2	2	2	2	2	2	0	2	0	2	2	2	0	0
NARO-HIST454		2000	2	1998(1);1999(1);	TARC(2);	0-0.5(0);0.5-1.	2	2	2	2	2	2	2	0	2	0	2	2	2	0	0
NARO-HIST455		2001	1	2000(1);	TARC(1);	0-0.5(0);0.5-1.	1	1	1	1	1	1	1	0	1	0	1	1	1	0	0
NARO-HIST456		2001	2	1999(1);2000(1);	TARC(2);	0-0.5(0);0.5-1.	2	2	2	1	1	2	2	0	2	0	2	2	2	0	0
NARO-HIST457		2001	2	1999(1);2000(1);	TARC(2);	0-0.5(0);0.5-1.	2	2	2	2	2	2	2	0	2	0	2	2	2	0	0
NARO-HIST458		2001	2	2000(1);2005(1);	TARC(2);	0-0.5(0);0.5-1.	2	1	1	2	2	2	2	0	2	0	2	2	2	0	0
NARO-HIST459		2001	2	1999(1);2000(1);	TARC(2);	0-0.5(0);0.5-1.	2	1	1	2	2	2	2	0	2	0	2	2	2	0	0
NARO-HIST460		2001	2	1999(1);2000(1);	TARC(2);	0-0.5(0);0.5-1.	2	2	2	2	2	2	2	0	2	0	2	2	2	0	0
NARO-HIST461		2002	13	2000(1);2008(1);	CARC(1);TARC(12);	0-0.5(0);0.5-1.	13	12	12	13	13	13	13	7	13	7	13	13	13	4	6
NARO-HIST462		2002	1	2004(1);	TARC(1);	0-0.5(0);0.5-1.	1	1	1	1	1	1	1	0	1	0	1	1	1	0	0
NARO-HIST463		2002	4	1999(1);2000(2);	CARC(2);TARC(2);	0-0.5(0);0.5-1.	4	4	4	4	4	4	4	4	4	4	4	4	4	0	0
NARO-HIST464		2003	23	2001(1);2002(1);	CARC(3);TARC(20);	0-0.5(0);0.5-1.	23	22	22	23	23	23	10	3	10	3	20	20	23	0	0
NARO-HIST465		2003	5	2002(1);2007(1);	TARC(5);	0-0.5(0);0.5-1.	5	5	5	5	5	5	5	5	5	5	5	5	5	0	0
NARO-HIST466		2004	2	2002(1);2003(1);	TARC(2);	0-0.5(0);0.5-1.	2	2	2	2	2	2	2	0	2	0	2	2	2	0	0
NARO-HIST467		2004	2	2002(1);2003(1);	TARC(2);	0-0.5(0);0.5-1.	2	2	2	2	2	2	2	0	2	0	2	2	2	0	0
NARO-HIST468		2004	3	2000(1);2002(1);	TARC(3);	0-0.5(0);0.5-1.	3	3	3	3	3	3	3	0	3	0	3	2	3	0	0
NARO-HIST469		2004	2	2003(2);	CARC(1);TARC(1);	0-0.5(0);0.5-1.	2	2	2	2	2	2	2	0	2	0	2	2	2	0	0
NARO-HIST470		2004	5	2002(1);2003(1);	TARC(5);	0-0.5(0);0.5-1.	5	5	5	5	5	5	0	2	0	0	3	2	5	0	0
NARO-HIST471		2004	1	2003(1);	TARC(1);	0-0.5(0);0.5-1.	1	1	1	1	1	1	0	0	0	0	1	1	1	0	0
NARO-HIST472		2004	7	2003(1);2008(2);	TARC(7);	0-0.5(0);0.5-1.	7	7	7	7	7	7	1	0	1	0	4	4	6	0	0
NARO-HIST473		2005	1	2004(1);	TARC(1);	0-0.5(0);0.5-1.	1	1	1	1	1	1	1	0	1	0	1	1	1	0	0
NARO-HIST474		2005	2	2003(1);2004(1);	TARC(2);	0-0.5(0);0.5-1.	2	2	2	2	2	2	2	0	2	0	2	2	2	0	0
NARO-HIST475		2005	3	2003(1);2004(2);	CARC(1);TARC(2);	0-0.5(0);0.5-1.	3	3	3	3	3	3	3	0	3	0	3	3	3	0	0
NARO-HIST476		2005	1	2004(1);	TARC(1);	0-0.5(0);0.5-1.	1	1	1	1	1	1	1	0	1	0	1	1	1	0	0
NARO-HIST477		2006	3	2003(1);2004(1);	TARC(3);	0-0.5(0);0.5-1.	3	3	3	3	3	3	3	0	3	0	3	3	3	0	0
NARO-HIST478		2006	1	2005(1);	TARC(1);	0-0.5(0);0.5-1.	1	0	0	1	1	1	1	0	1	0	1	1	1	0	0
NARO-HIST479		2007	2	2005(1);2006(1);	TARC(2);	0-0.5(0);0.5-1.	2	2	2	2	2	2	2	0	2	0	2	2	2	0	0
NARO-HIST480		2007	1	2006(1);	TARC(1);	0-0.5(0);0.5-1.	1	1	1	1	1	1	1	0	1	0	1	1	1	0	0
NARO-HIST481		2008	1	2007(1);	TARC(1);	0-0.5(0);0.5-1.	1	1	1	1	1	1	1	0	1	0	1	1	1	0	0
NARO-HIST482		2008	6	2007(1);2014(1);	TARC(6);	0-0.5(0);0.5-1.	6	6	6	6	6	6	6	5	6	5	6	6	6	3	4
NARO-HIST483		2009	1	2008(1);	TARC(1);	0-0.5(0);0.5-1.	1	1	1	1	1	1	1	0	1	0	1	1	1	0	0
NARO-HIST484		2009	7	2008(1);2014(1);	TARC(7);	0-0.5(0);0.5-1.	7	7	7	7	7	7	7	6	7	6	7	7	7	0	0
NARO-HIST485		2009	1	2008(1);	TARC(1);	0-0.5(0);0.5-1.	1	1	1	1	1	1	1	0	1	0	1	1	1	1	0
NARO-HIST486		2009	2	2008(1);2013(1);	TARC(2);	0-0.5(0);0.5-1.	2	2	2	2	2	2	1	0	1	0	2	2	2	0	0
NARO-HIST487		2009	1	2008(1);	TARC(1);	0-0.5(0);0.5-1.	1	1	1	1	1	1	0	0	0	0	1	1	1	0	0
NARO-HIST488		2010	3	2008(2);2009(1);	CARC(1);TARC(2);	0-0.5(0);0.5-1.	3	3	3	3	3	3	3	0	3	0	3	3	2	0	0
NARO-HIST489		2010	1	2009(1);	TARC(1);	0-0.5(0);0.5-1.	1	1	1	1	1	1	1	0	1	0	1	1	1	0	0
NARO-HIST490		2010	3	2009(1);2014(1);	TARC(3);	0-0.5(0);0.5-1.	3	3	3	3	3	3	0	0	0	0	1	2	3	0	0
NARO-HIST491		2011	2	2009(1);2010(1);	TARC(2);	0-0.5(0);0.5-1.	2	2	2	2	2	2	2	0	2	0	2	2	2	2	2
NARO-HIST492		2012	7	2009(1);2010(1);	TARC(7);	0-0.5(0);0.5-1.	7	7	7	7	7	7	7	4	7	4	7	7	7	4	5
NARO-HIST493		2012	2	2010(1);2011(1);	TARC(2);	0-0.5(0);0.5-1.	2	2	2	2	2	2	2	0	2	0	2	2	2	1	2
NARO-HIST494		2012	4	2010(1);2011(3);	CARC(1);TARC(3);	0-0.5(0);0.5-1.	4	4	4	4	4	4	0	0	0	0	4	4	4	0	0
NARO-HIST495		2012	4	2008(1);2009(1);	TARC(4);	0-0.5(0);0.5-1.	4	4	4	4	4	4	0	0	0	0	1	1	4	0	0
NARO-HIST496		2013	2	2011(1);2012(1);	TARC(2);	0-0.5(0);0.5-1.	2	2	2	2	2	2	2	0	2	0	2	2	2	1	2
NARO-HIST497		2013	8	2009(1);2010(2);	CARC(1);TARC(7);	0-0.5(0);0.5-1.	8	8	8	8	8	8	0	0	0	0	8	8	8	0	0
NARO-HIST498		2013	5	2007(1);2008(1);	CARC(1);TARC(4);	0-0.5(0);0.5-1.	5	5	5	5	5	5	5	5	5	5	5	5	5	0	0
NARO-HIST499		2013	4	2007(1);2008(1);	TARC(4);	0-0.5(0);0.5-1.	4	4	4	4	4	4	4	4	4	4	4	4	4	0	0
NARO-HIST500		2014	2	2012(1);2013(1);	TARC(2);	0-0.5(0);0.5-1.	2	2	2	2	2	2	2	0	2	0	2	2	2	1	1
NARO-HIST501		2014	2	2012(1);2013(1);	TARC(2);	0-0.5(0);0.5-1.	2	2	2	2	2	2	2	0	2	0	2	2	2	1	1
NARO-HIST502		2014	2	2012(1);2013(1);	TARC(2);	0-0.5(0);0.5-1.	2	2	2	2	2	2	2	0	2	0	2	2	2	1	1
NARO-HIST503		2014	2	2012(1);2013(1);	TARC(2);	0-0.5(0);0.5-1.	2	2	2	2	2	2	2	0	2	0	2	2	2	0	0
NARO-HIST504		2014	2	2010(1);2011(1);	TARC(2);	0-0.5(0);0.5-1.	2	2	2	2	2	2	2	2	2	2	2	2	2	2	2
NARO-HIST505		2015	2	2013(1);2014(1);	TARC(2);	0-0.5(0);0.5-1.	2	2	2	2	2	2	2	0	2	0	2	2	2	1	2
NARO-HIST506		2015	2	2013(1);2014(1);	TARC(2);	0-0.5(0);0.5-1.	2	2	2	2	2	2	2	0	2	0	2	2	2	1	2
NARO-HIST507		2015	2	2013(1);2014(1);	TARC(2);	0-0.5(0);0.5-1.	2	2	2	2	2	2	2	0	2	0	2	2	2	1	2
NARO-HIST508		2015	3	2012(1);2013(1);	TARC(3);	0-0.5(0);0.5-1.	3	3	3	3	3	3	3	0	3	0	3	3	3	0	0
NARO-HIST509		2015	3	2012(1);2013(1);	TARC(3);	0-0.5(0);0.5-1.	3	3	3	3	3	3	3	3	3	3	3	3	3	0	0
NARO-HIST510		2016	3	2013(1);2014(1);	TARC(3);	0-0.5(0);0.5-1.	3	3	3	3	3	3	3	3	3	3	3	3	3	1	2
NARO-HIST511		2016	3	2014(1);2015(2);	TARC(3);	0-0.5(0);0.5-1.	3	3	3	3	3	3	3	3	3	3	3	3	3	2	2
NARO-HIST512		2016	1	2014(1);	TARC(1);	0-0.5(0);0.5-1.	1	1	1	1	1	1	1	0	1	0	1	1	1	0	0
NARO-HIST513		2016	2	2014(1);2015(1);	TARC(2);	0-0.5(0);0.5-1.	2	2	2	2	2	2	2	0	2	0	2	2	2	0	0
NARO-HIST514		2016	2	2014(1);2015(1);	TARC(2);	0-0.5(0);0.5-1.	2	2	2	2	2	2	0	0	0	0	0	1	2	0	0
NARO-HIST515		2017	2	2015(1);2016(1);	TARC(2);	0-0.5(0);0.5-1.	2	2	2	2	2	2	2	2	2	2	2	2	2	1	2
NARO-HIST516		2017	2	2015(1);2016(1);	TARC(2);	0-0.5(0);0.5-1.	2	2	2	2	2	2	2	2	2	2	2	2	2	0	0
NARO-HIST517		2017	2	2015(1);2016(1);	TARC(2);	0-0.5(0);0.5-1.	2	2	2	2	2	2	2	2	2	2	2	2	2	1	2
NARO-HIST518		2018	3	2015(1);2016(1);	TARC(3);	0-0.5(0);0.5-1.	3	3	3	3	3	3	3	0	3	0	3	3	3	1	3
NARO-HIST519		2018	2	2016(1);2017(1);	TARC(2);	0-0.5(0);0.5-1.	2	2	2	2	2	2	2	2	2	2	2	2	2	0	0
NARO-HIST520		2019	2	2017(1);2018(1);	TARC(2);	0-0.5(0);0.5-1.	2	2	2	2	2	2	2	0	2	0	2	2	2	0	1
NARO-HIST521		2019	3	2017(1);2018(2);	CARC(1);TARC(2);	0-0.5(0);0.5-1.	3	3	3	3	3	3	3	0	3	0	3	3	3	0	0
NARO-HIST522		2009	4	2003(1);2004(1);	TARC(4);	0-0.5(0);0.5-1.	4	4	4	4	4	4	4	0	4	0	4	4	4	0	0
NARO-HIST523		2009	3	2003(1);2004(1);	TARC(3);	0-0.5(0);0.5-1.	3	3	3	3	3	3	3	0	3	0	3	3	3	0	0
NARO-HIST524		2010	3	2007(1);2008(1);	TARC(3);	0-0.5(0);0.5-1.	3	3	3	3	3	3	0	0	0	0	3	3	3	0	0
NARO-HIST525	NA		7	2013(1);2014(1);	TARC(7);	0-0.5(0);0.5-1.	7	7	7	7	7	7	7	7	7	7	7	7	7	0	0
NARO-HIST526		1966	11	1994(1);1995(1);	KARC(11);	0-0.5(0);0.5-1.	11	11	11	11	11	11	11	11	11	11	11	11	11	1	2
NARO-HIST527		1968	9	2004(1);2005(1);	KARC(9);	0-0.5(0);0.5-1.	9	9	9	9	9	9	9	9	9	9	9	8	9	0	2
NARO-HIST528		1970	20	1994(2);1997(2);	KARC(20);	0-0.5(0);0.5-1.	20	20	20	20	20	20	20	20	20	20	20	20	20	3	2
NARO-HIST529		1987	11	1995(1);1996(2);	KARC(11);	0-0.5(0);0.5-1.	11	11	11	11	11	11	10	10	10	10	10	10	11	5	0
NARO-HIST530		1992	16	2002(1);2007(2);	KARC(10);NICS(3);W	0-0.5(0);0.5-1.	16	15	15	16	16	16	14	16	14	14	16	16	16	5	4
NARO-HIST531		1992	7	2004(2);2005(2);	KARC(7);	0-0.5(0);0.5-1.	7	4	4	7	7	7	0	6	0	0	1	1	7	0	0
NARO-HIST532		1994	5	2003(2);2005(1);	NICS(4);WARC(1);	0-0.5(0);0.5-1.	5	5	5	5	5	5	5	5	5	5	5	5	5	0	0
NARO-HIST533		1996	1	1994(1);	KARC(1);	0-0.5(0);0.5-1.	1	1	1	1	1	1	1	1	1	1	1	1	1	1	1
NARO-HIST534		1996	1	1994(1);	KARC(1);	0-0.5(0);0.5-1.	1	1	1	1	1	1	1	1	1	1	1	1	1	1	1
NARO-HIST535		1996	1	1994(1);	KARC(1);	0-0.5(0);0.5-1.	1	1	1	1	1	1	1	1	1	1	1	1	1	1	1
NARO-HIST536		1996	1	1995(1);	KARC(1);	0-0.5(0);0.5-1.	1	1	1	1	1	1	1	1	1	1	1	1	1	0	1
NARO-HIST537		1997	49	1995(1);2006(1);	KARC(20);WARC(29)	0-0.5(0);0.5-1.	49	48	48	49	49	49	49	49	49	49	49	49	49	14	13
NARO-HIST538		1997	1	1994(1);	KARC(1);	0-0.5(0);0.5-1.	1	1	1	1	1	1	1	1	1	1	1	1	1	1	1
NARO-HIST539		1997	1	1996(1);	KARC(1);	0-0.5(0);0.5-1.	1	1	1	1	1	1	1	1	1	1	1	1	1	0	0
NARO-HIST540		1997	3	1994(1);1995(1);	KARC(3);	0-0.5(0);0.5-1.	3	3	3	3	3	3	3	3	3	3	3	3	3	0	0
NARO-HIST541		1997	1	1995(1);	KARC(1);	0-0.5(0);0.5-1.	1	1	1	1	1	1	1	1	1	1	1	1	1	1	1
NARO-HIST542		1998	1	1996(1);	KARC(1);	0-0.5(0);0.5-1.	1	1	1	1	1	1	1	1	1	1	1	1	1	0	0
NARO-HIST543		1998	3	1996(1);2004(1);	KARC(3);	0-0.5(0);0.5-1.	3	3	3	3	3	3	3	3	3	3	3	3	3	0	0
NARO-HIST544		1998	1	1996(1);	KARC(1);	0-0.5(0);0.5-1.	1	1	1	1	1	1	1	1	1	1	1	1	1	0	0
NARO-HIST545		1998	1	1996(1);	KARC(1);	0-0.5(0);0.5-1.	1	1	1	1	1	1	1	1	1	1	1	1	1	0	0
NARO-HIST546		1998	1	1996(1);	KARC(1);	0-0.5(0);0.5-1.	1	1	1	1	1	1	1	1	1	1	1	1	1	0	0
NARO-HIST547		1999	1	1997(1);	KARC(1);	0-0.5(0);0.5-1.	1	1	1	1	1	1	1	1	1	1	1	1	1	0	0
NARO-HIST548		1999	3	1997(1);1998(1);	KARC(3);	0-0.5(0);0.5-1.	3	3	3	3	3	3	3	3	3	3	3	3	3	0	0
NARO-HIST549		2000	2	1997(1);1999(1);	KARC(2);	0-0.5(0);0.5-1.	2	2	2	2	2	2	2	2	2	2	2	2	2	0	0
NARO-HIST550		2000	2	1998(1);1999(1);	KARC(2);	0-0.5(0);0.5-1.	2	2	2	2	2	2	2	2	2	2	2	2	2	0	0

NARO-HIST551		2001	2	1998(1);1999(1);	KARC(2);	0-0.5(0);0.5-1.	2	2	2	2	2	2	2	2	2	2	2	2	2	0	0
NARO-HIST552		2001	10	2007(1);2008(2);	KARC(10);	0-0.5(0);0.5-1.	10	10	10	10	10	10	10	10	10	10	10	10	10	5	0
NARO-HIST553		2002	1	2000(1);	KARC(1);	0-0.5(0);0.5-1.	1	1	1	1	1	1	1	1	1	1	1	1	1	0	0
NARO-HIST554		2002	47	2000(1);2001(1);	KARC(16);NICS(21);V	0-0.5(0);0.5-1.	45	46	45	46	46	46	46	43	46	43	46	46	46	8	7
NARO-HIST555		2003	3	2000(1);2001(1);	KARC(3);	0-0.5(0);0.5-1.	3	3	3	3	3	3	3	3	3	3	3	3	3	0	0
NARO-HIST556		2003	2	2001(1);2002(1);	KARC(2);	0-0.5(0);0.5-1.	2	2	2	2	2	2	2	2	2	2	2	2	2	0	0
NARO-HIST557		2003	15	2001(2);2002(3);	KARC(12);NICS(1);W	0-0.5(0);0.5-1.	15	7	7	15	14	14	1	12	1	0	6	6	15	0	0
NARO-HIST558		2004	1	2002(1);	KARC(1);	0-0.5(0);0.5-1.	1	1	1	1	1	1	1	1	1	1	1	1	1	0	0
NARO-HIST559		2004	2	2002(1);2003(1);	KARC(2);	0-0.5(0);0.5-1.	2	2	2	2	2	2	2	2	2	2	2	2	2	0	0
NARO-HIST560		2005	3	2002(1);2003(1);	KARC(3);	0-0.5(0);0.5-1.	3	3	3	3	3	3	3	3	3	3	3	2	3	0	0
NARO-HIST561		2005	4	2001(1);2002(1);	KARC(4);	0-0.5(0);0.5-1.	4	4	4	4	4	4	4	4	4	4	4	4	4	0	0
NARO-HIST562		2006	4	2004(1);2005(1);	KARC(4);	0-0.5(0);0.5-1.	4	4	4	4	4	4	4	4	4	4	4	4	4	2	2
NARO-HIST563		2006	3	2003(1);2004(1);	KARC(3);	0-0.5(0);0.5-1.	3	3	3	3	3	3	3	3	3	3	3	3	3	0	0
NARO-HIST564		2006	2	2004(1);2005(1);	KARC(2);	0-0.5(0);0.5-1.	2	2	2	2	2	2	2	2	2	2	2	2	2	0	0
NARO-HIST565		2006	9	2004(2);2005(3);	KARC(9);	0-0.5(0);0.5-1.	9	4	4	9	9	9	2	9	2	2	2	1	9	0	0
NARO-HIST566		2007	6	2004(2);2005(2);	KARC(6);	0-0.5(0);0.5-1.	6	4	4	6	6	6	0	6	0	0	1	1	6	0	0
NARO-HIST567		2007	4	2004(1);2005(1);	KARC(4);	0-0.5(0);0.5-1.	4	2	2	4	4	4	3	4	3	3	3	2	4	0	0
NARO-HIST568		2007	1	2006(1);	KARC(1);	0-0.5(0);0.5-1.	1	0	0	0	0	0	0	0	0	0	0	0	1	0	0
NARO-HIST569		2008	3	2005(1);2006(1);	KARC(3);	0-0.5(0);0.5-1.	3	3	3	3	3	3	3	3	3	3	3	3	3	0	0
NARO-HIST570		2008	2	2006(1);2007(1);	KARC(2);	0-0.5(0);0.5-1.	2	2	2	2	2	2	2	2	2	2	2	2	2	0	0
NARO-HIST571		2009	2	2007(1);2008(1);	KARC(2);	0-0.5(0);0.5-1.	2	2	2	2	2	2	2	2	2	2	2	2	2	1	0
NARO-HIST572		2009	2	2007(1);2008(1);	KARC(2);	0-0.5(0);0.5-1.	2	2	2	2	2	2	2	2	2	2	2	2	2	1	0
NARO-HIST573		2009	2	2007(1);2008(1);	KARC(2);	0-0.5(0);0.5-1.	2	2	2	2	2	2	2	2	2	2	2	2	2	1	0
NARO-HIST574		2009	6	2006(1);2007(1);	KARC(6);	0-0.5(0);0.5-1.	6	6	6	6	6	6	6	6	6	6	6	6	6	1	0
NARO-HIST575		2010	2	2008(1);2009(1);	KARC(2);	0-0.5(0);0.5-1.	2	2	2	2	2	2	2	2	2	2	2	2	2	2	0
NARO-HIST576		2010	5	2008(1);2009(1);	KARC(5);	0-0.5(0);0.5-1.	5	5	5	5	5	5	5	5	5	5	5	5	5	2	0
NARO-HIST577		2011	3	2008(1);2009(1);	KARC(3);	0-0.5(0);0.5-1.	3	3	3	3	3	3	3	3	3	3	3	3	3	3	0
NARO-HIST578		2011	2	2009(1);2010(1);	KARC(2);	0-0.5(0);0.5-1.	2	2	2	2	2	2	2	2	2	2	2	2	2	2	0
NARO-HIST579		2011	3	2008(1);2009(1);	KARC(3);	0-0.5(0);0.5-1.	3	3	3	3	3	3	3	3	3	3	3	3	3	3	0
NARO-HIST580		2011	3	2008(1);2009(1);	KARC(3);	0-0.5(0);0.5-1.	3	3	3	3	3	3	3	3	3	3	3	3	3	0	0
NARO-HIST581		2011	6	2008(1);2009(1);	KARC(4);NICS(1);WA	0-0.5(0);0.5-1.	6	1	1	6	6	6	1	4	1	0	1	1	6	0	0
NARO-HIST582		2011	3	2009(1);2010(2);	KARC(3);	0-0.5(0);0.5-1.	3	0	0	3	3	3	0	3	0	0	0	0	3	0	0
NARO-HIST583		2011	2	2009(1);2010(1);	KARC(2);	0-0.5(0);0.5-1.	2	2	2	2	2	2	2	2	2	2	2	2	2	2	0
NARO-HIST584		2012	3	2009(1);2010(1);	KARC(3);	0-0.5(0);0.5-1.	3	3	3	3	3	3	3	3	3	3	3	3	3	3	3
NARO-HIST585		2012	2	2010(1);2011(1);	KARC(2);	0-0.5(0);0.5-1.	2	2	2	2	2	2	2	2	2	2	2	2	2	2	2
NARO-HIST586		2012	3	2009(1);2010(1);	KARC(3);	0-0.5(0);0.5-1.	3	3	3	3	3	3	3	3	3	3	3	3	3	3	3
NARO-HIST587		2012	3	2009(1);2010(1);	KARC(3);	0-0.5(0);0.5-1.	3	3	3	3	3	3	3	3	3	3	3	3	3	0	2
NARO-HIST588		2012	4	2010(2);2011(2);	KARC(4);	0-0.5(0);0.5-1.	4	0	0	4	4	4	0	4	0	0	0	0	4	0	0
NARO-HIST589		2013	2	2011(1);2012(1);	KARC(2);	0-0.5(0);0.5-1.	2	2	2	2	2	2	2	2	2	2	2	2	2	2	2
NARO-HIST590		2013	2	2011(1);2012(1);	KARC(2);	0-0.5(0);0.5-1.	2	2	2	2	2	2	2	2	2	2	2	2	2	2	2
NARO-HIST591		2013	2	2011(1);2012(1);	KARC(2);	0-0.5(0);0.5-1.	2	2	2	2	2	2	2	2	2	2	2	2	2	2	2
NARO-HIST592		2013	2	2011(1);2012(1);	KARC(2);	0-0.5(0);0.5-1.	2	2	2	2	2	2	2	2	2	2	2	2	2	2	2
NARO-HIST593		2013	3	2011(2);2012(1);	KARC(3);	0-0.5(0);0.5-1.	3	3	3	3	3	3	3	3	3	3	3	3	3	0	0
NARO-HIST594		2014	2	2013(1);2014(1);	KARC(2);	0-0.5(0);0.5-1.	2	2	2	2	2	2	2	2	2	2	2	2	2	0	2
NARO-HIST595		2014	3	2012(1);2013(1);	KARC(3);	0-0.5(0);0.5-1.	3	3	3	3	3	3	3	3	3	3	3	3	3	0	2
NARO-HIST596		2014	3	2012(1);2013(1);	KARC(3);	0-0.5(0);0.5-1.	3	3	3	3	3	3	3	3	3	3	3	3	3	0	2
NARO-HIST597		2015	2	2014(1);2015(1);	KARC(2);	0-0.5(0);0.5-1.	2	2	2	2	2	2	2	2	2	2	2	2	2	0	0
NARO-HIST598		2015	3	2013(1);2014(1);	KARC(3);	0-0.5(0);0.5-1.	3	3	3	3	3	3	3	3	3	3	3	3	3	2	3
NARO-HIST599		2017	2	2015(1);2016(1);	KARC(2);	0-0.5(0);0.5-1.	2	2	2	2	2	2	2	2	2	2	2	2	2	0	0
NARO-HIST600		2017	2	2015(1);2016(1);	KARC(2);	0-0.5(0);0.5-1.	2	2	2	2	2	2	2	2	2	2	2	2	2	0	0
NARO-HIST601		2018	2	2016(1);2017(1);	KARC(2);	0-0.5(0);0.5-1.	2	2	2	2	2	2	2	2	2	2	2	2	2	2	2
NARO-HIST602		2017	4	2016(1);2017(3);	KARC(4);	0-0.5(0);0.5-1.	4	4	4	4	4	4	4	4	4	4	4	4	4	2	2
NARO-HIST603		2018	2	2016(1);2017(1);	KARC(2);	0-0.5(0);0.5-1.	2	2	2	2	2	2	2	2	2	2	2	2	2	0	0
NARO-HIST604		2018	2	2016(1);2017(1);	KARC(2);	0-0.5(0);0.5-1.	2	2	2	2	2	2	2	2	2	2	2	2	2	0	0
NARO-HIST605		2019	5	2016(1);2017(2);	KARC(4);WARC(1);	0-0.5(0);0.5-1.	5	5	5	5	5	5	5	4	5	4	5	5	5	0	0
NARO-HIST606	NA		2	2017(1);2018(1);	KARC(2);	0-0.5(0);0.5-1.	2	2	2	2	2	2	2	2	2	2	2	2	2	0	0
NARO-HIST607		2002	2	2001(1);2002(1);	KARC(2);	0-0.5(0);0.5-1.	2	2	2	2	2	2	2	2	2	2	2	2	2	0	0
NARO-HIST608		1958	3	1993(1);1994(1);	WARC(3);	0-0.5(0);0.5-1.	3	3	3	3	3	3	3	3	3	3	3	3	3	0	0
NARO-HIST609		2009	1	2016(1);	KARC(1);	0-0.5(0);0.5-1.	1	1	1	1	1	1	1	1	1	1	1	1	1	0	0
NARO-HIST610		1981	2	2001(1);2002(1);	HARC(2);	0-0.5(0);0.5-1.	2	2	2	2	2	2	2	1	2	1	2	2	2	1	1
NARO-HIST611		1990	20	1997(2);1998(2);	HARC(20);	0-0.5(0);0.5-1.	14	14	14	14	14	14	14	14	14	14	14	14	14	7	15
NARO-HIST612		1993	2	2001(1);2002(1);	HARC(2);	0-0.5(0);0.5-1.	2	2	2	2	2	2	2	2	2	2	2	2	2	1	1
NARO-HIST613		1998	53	2003(1);2006(2);	HARC(53);	0-0.5(3);0.5-1.	45	43	43	45	45	45	33	44	33	33	43	36	45	34	36
NARO-HIST614		1984	6	1999(2);2000(2);	NICS(6);	0-0.5(0);0.5-1.	6	6	6	6	6	6	2	6	2	2	2	1	6	0	0
NARO-HIST615		1960	2	1997(1);1998(1);	TARC(2);	0-0.5(0);0.5-1.	2	2	2	2	2	2	2	0	2	0	2	2	2	0	0
NARO-HIST616		1988	150	1994(1);1995(2);	CARC(63);NICS(26);T	0-0.5(0);0.5-1.	150	149	149	150	150	150	140	100	140	100	148	148	149	20	26
NARO-HIST617		1991	3	1999(2);2000(1);	TARC(3);	0-0.5(0);0.5-1.	3	3	3	3	3	3	3	0	3	0	3	3	3	0	0
NARO-HIST618		1995	1	2000(1);	TARC(1);	0-0.5(0);0.5-1.	1	1	1	1	1	1	1	0	1	0	1	1	1	0	0
NARO-HIST619		2009	2	2013(1);2014(1);	TARC(2);	0-0.5(0);0.5-1.	2	2	2	2	2	2	2	0	2	0	2	2	2	0	0
NARO-HIST620		1999	7	2009(1);2010(4);	HARC(7);	0-0.5(0);0.5-1.	7	7	7	7	7	7	7	7	7	7	7	7	7	7	7
NARO-HIST621		1990	7	1997(1);1998(1);	TARC(7);	0-0.5(0);0.5-1.	7	7	7	7	7	7	7	2	7	2	7	7	7	0	0
NARO-HIST622		2001	2	2008(1);2011(1);	TARC(2);	0-0.5(0);0.5-1.	1	1	1	1	1	1	1	0	1	0	1	1	1	2	1
NARO-HIST623	NA		4	1997(1);1998(1);	CARC(4);	0-0.5(0);0.5-1.	4	4	4	4	4	4	4	4	4	4	4	4	4	0	0
NARO-HIST624	NA		1	2006(1);	TARC(1);	0-0.5(0);0.5-1.	1	1	1	1	1	1	1	0	1	0	1	1	1	0	0
NARO-HIST625	NA		1	2006(1);	KARC(1);	0-0.5(0);0.5-1.	1	0	0	0	0	0	0	0	0	0	0	0	1	0	0
NARO-HIST626	NA		4	2008(1);2009(1);	TARC(4);	0-0.5(0);0.5-1.	4	4	4	4	4	4	0	0	0	0	1	1	4	0	0

[a] Numbers in brackets represent the data count.

Supplemental Table 6. Selected fixed effects, random effects, and their interactions examined by using the step function of R-package lmerTest

	Random effect		Fixed effect		Interaction										
	G	Y	L	F	GY	GL	GF	YL	YF	LF	GYL	GYF	GLF	YLF	GYLF
Days to heading	✓	✓	✓	✓		✓		✓		✓	✓		✓	✓	
Days to maturity	✓	✓	✓	✓		✓	✓	✓		✓	✓			✓	
Ripening period	✓	✓	✓	✓		✓	✓	✓		✓	✓			✓	
Culm length	✓	✓	✓	✓	✓	✓		✓	✓	✓	✓		✓	✓	
Panicle length	✓		✓	✓		✓		✓			✓		✓	✓	✓
Number of panicles	✓	✓	✓	✓		✓		✓	✓	✓	✓		✓	✓	
Number of grains per panicle	✓	✓	✓	✓	✓	✓		✓		✓	✓	✓	✓	✓	
Plant weight	✓	✓	✓	✓		✓	✓	✓	✓	✓	✓			✓	
Grain yield	✓		✓	✓		✓	✓	✓			✓			✓	
Harvest index	✓	✓	✓	✓		✓	✓	✓	✓	✓	✓			✓	
Thousand-grain weight	✓		✓	✓		✓		✓		✓	✓		✓	✓	
Grain quality	✓	✓	✓	✓	✓	✓		✓			✓			✓	
Lodging degree	✓	✓	✓	✓	✓	✓		✓	✓	✓	✓		✓	✓	
Total	13	10	13	13	4	13	5	13	5	10	13	1	7	13	1

G, genotype; Y, year; L, location; F, manure level
